# Supplementary material for: Efficacy of Wang Nam Yen herbal tea on human milk production: A randomized controlled trial
Source: PLoS One. 2022 Jan 31;17(1):e0247637. doi: 10.1371/journal.pone.0247637 (PMC8803155; doi:10.1371/journal.pone.0247637)
Supplement: S2 File — (DOCX) [file pone.0247637.s003.docx]

**Trial Protocol Synopsis**

“Efficacy of Wang Nam Yen Herbal Tea on Human Milk Production: A Randomized Placebo Controlled Trial (Tea4Milk)”

Koollachart Saejueng, MD^1^

Piyawadee Wuttikonsammakit, MD^1^

Wattanaporn Khumbun, MD^2^
Krit Pongpirul, MD, MPH, PhD^3^

^1^Department of Obstetrics and Gynecology, Sunpasitthiprasong Hospital, Ubon Ratchathani, Thailand

^2^Department of Thai Traditional and Alternative Medicine, Sunpasitthiprasong Hospital,

Ubon Ratchathani, Thailand
^3^Department of Preventive and Social Medicine, Faculty of Medicine, Chulalongkorn University, Bangkok, Thailand

**Contents**

Page

Contents….……………………………………………………………………….. 2

Introduction.………………………………… …………………………………….. 3

Methods………… ……………………………………………………………….. 4

- Study design……………………………………………………………… 4
- Study population…… …………………………………………………… 4
- Inclusion criteria…………….………………………………………….... 4
- Exclusion criteria………………. ……………………………………….. 4
- Sample size calculation…………………………………………………... 5
- Randomization…......................................................................................... 6
- Blinding………………............................................................................... 7
- Outcome measures…..…………………………………………………… 8
- Trial procedure.…………………………………………………………. 8

Statistical analysis………. ……………………………………………………... 11

Timetable………..…………………………………………………………......... 13

References.. ………………………………………………………………..…... 14

**Introduction**

Since the ancient Thailand, postpartum women sought for lactation stimulating foods and herbs from Thai and Chinese medicine to match their babies’ needs. There were studies outside of Thailand on domperidone that proved its the galactagogue effect. Domperidone was the only galactagogue that had efficacy and safety evidence supported by randomized controlled trials.^1^ However, U.S. Food and Drug Administration (USFDA) recently warned for intravenous form of domperidone.^2,3^ There was no evidence on adverse events for oral form of domperidone.^4^ Thai traditional herbal medicine had potential to be an alternative galactagogue. In Thai traditional medicine, there were several purposes for postpartum herbal uses including promote lochia discharge, blood clot discharge, fundal progression, physical health and lactation. Most herbs for postpartum had acrid properties which promote fire element in the body according to Thai traditional medicine wisdom which stated that human body was comprised of four fundamental elements: earth, water, fire, and wind. A healthy state could be maintained by balance of four fundamental elements while illness or disease was caused by elemental imbalance. Postpartum women lost stamina, water, and blood from delivery which caused imbalance of water, fire and wind elements. Acrid herbs alleviated fire element which promote recovery organ systems in human body.^5^

There were world-wide practices of using herbs for promoting lactation.^6^ Although there was lack of evidence on mechanism of actions, herbal medicine was widely used, varied by traditional wisdom and cultures, had evidence on efficacy and no evidence on major adverse events. There was scarce of study on Thai traditional herbal medicine on promoting lactation. There was only a grey literature on lactation promoting tea consisted of sappan (Caesalpinia sappan Linn.), licorice (Glycyrrhiza glabra Linn.), bale fruit (Aegle marmelos L. Corr), ginger (Zingiber officinale Roscoe), and jewel vine (Glycyrrhiza glabra Linn.), which had lactation promoting, physical promoting, and muscle pain reducing properties, for postpartum women to reduce neonatal jaundice from inadequate breastfeeding. There was reduction on incidence of neonatal jaundice from 10% to 3% and all postpartum women had breast milk production in 24 hours postpartum.^7^

The Wang Nam Yen herbal tea was a promising traditional Thai galactagogue, which might be used as an alternative to the common pharmaceutical galactagogue, domperidone, to support breastfeeding difficulties due to low supply. Domperidone was used as active control with dosage 30 mg/day which evidence proved no difference in human milk production with higher dosage^8^. Women that have undergone cesarean deliveries was selected as population in this study due to cesarean delivery was a risk factor of inadequate breast milk production.^9^

**Methods**

**Study design**

This study is a randomized double blind controlled trial.

**Study population**

Women aged 15 to 41 years who deliver via cesarean section at 28 to 42 weeks’ gestational age.

**Study sample**

Women aged 15 to 41 years who deliver via cesarean section at 28 to 42 weeks’ gestational age at Sunpasithiprasong Hospital, Ubon Ratchathani, Thailand during February 2017 to September 2017.

**Inclusion criteria**

Women aged 15 to 41 years who deliver via cesarean section at 28 to 42 weeks’ gestational age at Sunpasithiprasong Hospital, Ubon Ratchathani, Thailand during February 2017 to September 2017 and provide written informed consent.

**Exclusion criteria**

1. Women who have contraindications for breastfeeding such as human immunodeficiency virus infection
2. Women who have serious peripartum complications leading to inability for breastfeeding such as severe postpartum hemorrhage with hypovolemic shock, cardiac arrest, severe preeclampsia and eclampsia
3. Women with history of domperidone or tea herbal ingredients allergy
4. Women whose neonates are sick and separated from mother

**Sample size calculation**

We use mean and standard deviation of breast milk volume at day 4 after delivery based on the study by based on the study by Jantarasaengaram and Sreewapaon^10^ the effects of domperidone compared to placebo in augmenting lactation following cesarean delivery with 80% power and a 2-sided type I error at 5%

Mean daily milk volume in the domperidone group day 4= 191.3(SD=136.1)

Mean daily milk volume in the placebo group day4 = 91.4 (SD=60.3)

n/group = $\frac{3{(Z\alpha+Z\beta)}^{2}\delta^{2}}{\left( \mu_{1}-\mu_{2} \right)^{2}}$

$\delta=SD of primary outcome$

$$\mu_{1}=191.3 (case group)$$

$$\mu_{2} =91.4 (control group)$$

Consider α = 0.05, Zα = 1.65 Power = 80% Zβ = 0.84

n/group = $\frac{3\left( 1.65+0.84 \right)^{2}{(136.1)}^{2}}{{(191.3-91.4)}^{2}}$

Resulted in n/group = 34.52

With considering an expected follow-up loss of 15%, the anticipated sample size of 120 which would require a sample size of 40 in each group.

1. T– The Wang Nam Yen herbal tea treatment group

2. D– The domperidone treatment group

3. C– The placebo control group

**Randomization**

Participants will be random allocated into 3 groups with 1 : 1 : 1 ratio by block randomization with block of three using table of random numbers for creating randomization ID which will be enveloped in opaque envelop to mask clinicians, data collectors and patients.

Randomization ID will be assigned as

A = T– The Wang Nam Yen herbal tea treatment group which will receive Wang Nam Yen herbal tea and placebo tablet after each meal for three meals per day.

B = D– The domperidone treatment group which will receive herbal tea placebo and a 10 mg domperidone tablet after each meal for three meals per day.

C = C– The placebo control group which will recieve both herbal tea placebo and a placebo tablet after each meal for three meals per day.

There will be 6 possible blocks of three including 1 = ABC, 2 = ACB, 3 = BAC, 4 = BCA, 5 = CAB และ 6 = CBA

When 120 randomization ID are generated, the ID will be joined together starting with randomization ID for participant number 9 (TD009) as the first ID.

**Blinding**

After generating randomization codes, the research team which responsible for blinding will create opaque envelope to seal the randomization codes. The front end of an opaque sealed envelope will present study name, principal investigator name, site of study, randomization ID, study ID, and date which envelope will be opened (Figure 1).

Study : Efficacy of Wang Nam Yen Herbal Tea on Breast Milk Production : A Factorial Randomized Controlled Trial (Tea4Milk)

PI : Koollachart Saejueng

Site : Sunpasitthiprasong hospital

Randomization ID : TD001

Study ID : ________________________________________

Date envelope opened : ____________________________

**Figure 1.** Example of the front end of an opaque sealed envelope

There will be a document inside the sealed envelope with randomization ID, assigned group, study ID, date and time which envelope will be opened, and signature of envelope opener who are not clinician nor data collector (Figure 2).

Randomization ID : TD001

Assessment group :

Study ID : ________________________________________

Date envelope opened : ____________________________

Time envelope opened : ____________________________

Signature the person who randomizing the subject :

________________________________________________

**Figure 2.** Example of the document inside an opaque sealed envelope

**Outcome measures**

The primary outcome was breast milk volume at 72 hours after delivery. All participants were encouraged to breastfeed their infant within 24 hours of delivery based on Sanpasitthiprasong hospital's standard of care policy of early breastfeeding, frequent breastfeeding, correct positioning and avoiding the use of formula when possible. The breast milk volume was measured with a two-hour interval from last breastfeeding, using an electronic pump (Spectra S2Plus^®^, Spectra Baby USA). The electronic pump was applied for 15 minutes on each breast and the total volume of milk was recorded. Breast milk volume was also measured at 24 hours and 48 hours post-delivery. The breast milk volume at 24 hours post-delivery was used as baseline.

Secondary outcomes were observed during 72 hours after delivery period including:

(1) pregnancy outcomes such as postpartum hemorrhage, endometritis.

(2) neonatal outcomes such as neonatal jaundice, respiratory distress.
(3) adverse effects including headache, dry mouth, diarrhea, muscle cramps, itching, or allergic reactions.

(4) participant satisfaction during the three days’ postpartum period were also recorded.

**Trial procedure**

1. Literature review for relevant studies.
2. Drafting trial protocol and design case record form for ethical committee approval.
3. Plan for education for standard postpartum care and breastfeeding to women aged

15 to 41 years who deliver via cesarean section at 28 to 42 weeks’ gestational age at Sunpasithiprasong Hospital, Ubon Ratchathani, Thailand during February 2017 to

September 2017 and provide written informed consent.

1. Recruiting participants according to inclusion and exclusion criteria by placing advertisements in four obstetrics wards.
2. Giving trial information to potential participants using obstetrician and nurse in each obstetric ward and participant information sheet to confirm participant understanding of trial objective, procedure, benefits, and risks. If potential participants are willing to participate in trial, they will be asked to sign informed consent form with understanding that they can quit from trial at any time without any consequence.
3. Participants will be randomly assigned into three groups based on numbers generated by a table of random numbers in blocks of three: 1) Wang Nam Yen herbal tea and placebo tablet (T group), 2) domperidone tablet and placebo herbal tea (D group), 3) placebo tablet and placebo herbal tea (C group).
4. All study interventions will be prepacked and sealed in opaque packages, for three consecutive days of postpartum use. Participants and investigators were blinded to the treatment assignment. Randomization ID will be TD001 to TD120.
5. Wang Nam Yen herbal tea will be manufactured by Wang Nam Yen hospital, a community hospital in Sa Kaeo reputed for Thai traditional herbal medicine. Each Wang Nam Yen herbal tea contains sappan (*Caesalpinia sappan* Linn.) 500 mg, licorice (*Glycyrrhiza glabra* Linn.) 500 mg, bale fruit (*Aegle marmelos* L. Corr) 500 mg, ginger (*Zingiber officinale* Roscoe) 500 mg, and jewel vine (*Glycyrrhiza glabra* Linn.) 500 mg. Wang Nam Yen herbal tea will be packed in tea bag (2500 mg) designed for each meal. The herbal tea placebo will be prepared as tea bag for each meal, and the placebo tea bag will be identical with Wang Nam Yen tea bag. Both herbal teas will be prepared by diffusion in 200 mL of warm water for 5 minutes. Interventions will be administered orally three times per day after meals. The first administration will occur 12-18 hours following delivery to ensure sufficient time for the evaluation of any postpartum complications that may have occurred. Domperidone will be administered at 10 mg per dose, as 30 mg of domperidone per day are considered safe and sufficient for increasing breast milk production. Placebo tablets are identical to appearance of domperidone tablet. All participants (T, D, C groups) will be administered 200 mL of tea and a tablet after each meal.

T– The Wang Nam Yen herbal tea treatment group will receive Wang Nam Yen herbal tea and placebo tablet after each meal for three meals per day. This is an intervention arm which is designed to assess the efficacy of Wang Nam Yen herbal tea.

D– The domperidone treatment group will receive herbal tea placebo and a 10 mg domperidone tablet after each meal for three meals per day. This is an active control arm which is designed to be comparator as standard treatment.
C– The placebo control group will receive both herbal tea placebo and a placebo tablet after each meal for three meals per day. This is a placebo control arm which is designed to be a reference group with no treatment.

1. Demographic data will be recorded including age, occupation, monthly income, method of health payment, highest education. Obstetric data will be recorded including gestational age, numbers of pregnancy, numbers of delivery, numbers of antenatal visits for this pregnancy, presence of labor symptoms, indication of cesarean delivery, experience of breast feeding, nurturing environment, baby birthweight, sex of baby, immediate postpartum complication, fluid intake and output, and neonatal complication.
2. Participants will be asked to collect breast milk after received intervention at 24-72 hours postpartum using automated electric breast pump brand spectra type spectra 2 which has minimal pressure at 10 mmHg and maximum pressure at 300 mmHg. Pump cycle is approximately 42 times per minute. Breast pump will be 2 hours after last breastfeeding and pump for 15 minutes each breast. Breast milk will be recorded as milliliters (ml).
3. Drug adverse events including dry mouth, headache, insomnia, abdominal pain, diarrhea, nausea and urinary retention will be recorded.
4. Fluid intake and output will be recorded. Numbers of breastfeeding per day will be recorded.
5. Changes of bodyweight of mother and neonate will be recorded.
6. Before discharge, participants will be recorded for vital signs, fundal height, lochia color, postpartum complication, neonatal complication and participant satisfaction.
7. Collected data will be analyzed.
8. Result will be reported as standard format.
9. Conclusion, discussion and manuscript preparation.

**Statistical analysis**

The statistical analysis will be performed on an intention-to-treat basis using Stata/MP software version 15.0 (StataCorp 2017, College Station, TX). Descriptive statistics will be carried out using mean with standard deviation for normal distributed continuous data, and median with interquartile range for non-normal distributed continuous data. Categorical data will be presented as counts and percentage and will be tested for significance with Chi-square test. Continuous data will be assessed for normal distribution using histogram and Shapiro–Wilk test. One-way ANOVA with Bonferroni correction will be used for normal distributed continuous data and Kruskall-Wallis test will be used for non-normal distributed continuous data. Significant level is defined as *p*<0.05.

**Figure 3.** Trial procedure

Placing advertisements in four obstetrics wards

Recruiting participant eligible for inclusion and exclusion criteria

Obtaining informed consent from participants

Randomization into 3 groups

| T (n=40)  Wang Nam Yen herbal tea and placebo tablet | D (n=40)  herbal tea placebo and domperidone tablet | C (n=40)  herbal tea placebo and a placebo tablet |
| --- | --- | --- |

Recording baseline data and first administration of intervention at 12-18 hours postpartum

Recording fluid intake and output, numbers of breastfeeding per day, breast milk volume and
drug adverse events at 24, 48 and 72 hours postpartum

Recording primary and secondary outcomes at 72 hours postpartum

**Time table**

| **Trial procedure** | **Jun.2016** | **Jul.2016** | **Aug.2016** | **Sep.2016** | **Oct.2016** | **Nov.2016** | **Dec.2016** | **Jan.2017** | **Feb-Sep.2017** | | **Oct.2017** | **Nov.2017** | **Dec.2017** |
| --- | --- | --- | --- | --- | --- | --- | --- | --- | --- | --- | --- | --- | --- |
| Literature review |  |  |  |  |  |  |  |  |  |  |  |  |  |
| Protocol drafting |  |  |  |  |  |  |  |  |  |  |  |  |  |
| Proposal approval from obstetric department |  |  |  |  |  |  |  |  |  |  |  |  |  |
| Proposal approval from ethical committee |  |  |  |  |  |  |  |  |  |  |  |  |  |
| Trial information explanation |  |  |  |  |  |  |  |  |  |  |  |  |  |
| Data collection |  |  |  |  |  |  |  |  |  |  |  |  |  |
| Data analysis |  |  |  |  |  |  |  |  |  |  |  |  |  |
| Manuscript drafting |  |  |  |  |  |  |  |  |  |  |  |  |  |
| Manuscript editing |  |  |  |  |  |  |  |  |  |  |  |  |  |
| Manuscript presentation |  |  |  |  |  |  |  |  |  |  |  |  |  |

**References**

1. Osadchy A, Moretti ME, Koren G. Effect of domperidone on insufficient lactation in puerperal women: a systematic review and meta-analysis of randomized controlled trials. ObstetGynecol Int. 2012; 2012: 642893.
2. Johannes CB, Varas-Lorenzo C, McQuay LJ, Midkiff KD, Fife D. Risk of seriousventricular arrhythmia and sudden cardiac death in a cohort of usersof domperidone: a nested case-control study. Pharmacoepidemiol Drug Saf2010;19:881-8.
3. Van Noord C, Dieleman JP, van Herpen G, Verhamme K, Sturkenboom MC.Domperidone and ventricular arrhythmia or sudden cardiac death: a populationbasedcase-control study in the Netherlands. Drug Saf2010; 33: 1003-14.
4. Zuppa AA, Sindico P, Orchi C, Carducci C, Cardiello V, Romagnoli C, et al. Safety and efficacy of galactogogues: substances that induce, maintain and increase breast milk production. J Pharm PharmaceutSci2010; 13:162-74.
5. Termwiset P. Postpartum care in Thai traditional medicine. M.P.T: War veterans organization of Thailand press. 2^nd^ edition.; 2009:103-104.
6. Budzynska K, Gardner ZE, Dugoua JJ, Low Dog T, Gardiner P. Systematic review of breastfeeding and herbs. Breastfeed Med 2012; 7: 489-503.
7. Surakarn J, Chinsoy P, Chamnanaksorn W, Supaka N, Ngamwong S. Efficacy of breast milk stimulating herbal tea on human milk volume and serum prolactin level in postpartum women. 2011. (unpublished data)
8. [Wan EW](http://www.ncbi.nlm.nih.gov/pubmed/?term=Wan%20EW%5BAuthor%5D&cauthor=true&cauthor_uid=18507654), [Davey K](http://www.ncbi.nlm.nih.gov/pubmed/?term=Davey%20K%5BAuthor%5D&cauthor=true&cauthor_uid=18507654), [Page-Sharp M](http://www.ncbi.nlm.nih.gov/pubmed/?term=Page-Sharp%20M%5BAuthor%5D&cauthor=true&cauthor_uid=18507654), [Hartmann PE](http://www.ncbi.nlm.nih.gov/pubmed/?term=Hartmann%20PE%5BAuthor%5D&cauthor=true&cauthor_uid=18507654), [Simmer K](http://www.ncbi.nlm.nih.gov/pubmed/?term=Simmer%20K%5BAuthor%5D&cauthor=true&cauthor_uid=18507654), [Ilett KF](http://www.ncbi.nlm.nih.gov/pubmed/?term=Ilett%20KF%5BAuthor%5D&cauthor=true&cauthor_uid=18507654). Dose-effect study of domperidone as a galactagogue in preterm mothers with insufficient milk supply, and its transfer into milk.[Br J ClinPharmacol.](http://www.ncbi.nlm.nih.gov/pubmed/18507654) 2008;66:283-9.
9. Pierro J, Abulaimoun B, Roth P, Blau J. Factors associated with supplemental formula feeding of breastfeeding infants during postpartum hospital stay. Breastfeed Med 2016; 11: 196-202.
10. Jantarasaengaram S, Sreewapa P. Effect of domperidone on augmentation of lactation following cesarean delivery at full term:a randomized, double-blind, placebo-controlled trial.International Journal of Gynecology and obstestrics 2012; 116:240-243
