# Supplementary material for: Efficacy of Wang Nam Yen herbal tea on human milk production: A randomized controlled trial
Source: PLoS One. 2022 Jan 31;17(1):e0247637. doi: 10.1371/journal.pone.0247637 (PMC8803155; doi:10.1371/journal.pone.0247637)
Supplement: S3 File — (DOCX) [file pone.0247637.s004.docx]

**ข้อเสนอโครงการวิจัย**

**เรื่อง** ประสิทธิผลของชาสมุนไพรบำรุงน้ำนม (รพ.วังน้ำเย็น) ในการกระตุ้นการผลิตน้ำนมมารดาหลังคลอดบุตร

“Efficacy of Wang Nam Yen Herbal Tea on Human Milk Production: A Randomized Placebo Controlled Trial (Tea4Milk)”

นายแพทย์กุลชาติ แซ่จึง

Koollachart Saejueng,MD^1^

แพทย์หญิงปิยวดี วุฒิกรสัมมากิจ

Piyawadee Wuttikonsammakit, MD^1^

แพทย์หญิงวัฒนาพร คุ้มบุญ
Wattanaporn Khumbun, MD^2^

นายแพทย์กฤษณ์ พงศ์พิรุฬห์
Krit Pongpirul, MD, MPH, PhD^3^

^1^ กลุ่มงานสูตินรีเวชกรรม โรงพยาบาลสรรพสิทธิประสงค์ จังหวัดอุบลราชธานี

^2^กลุ่มงานแพทย์แผนไทยและการแพทย์ทางเลือก โรงพยาบาลสรรพสิทธิประสงค์ จังหวัดอุบลราชธานี

^3^ภาควิชาเวชศาสตร์ป้องกันและสังคม คณะแพทยศาสตร์ จุฬาลงกรณ์มหาวิทยาลัย กรุงเทพมหานคร

**สารบัญเรื่อง**

หน้า

สารบัญเรื่อง……………………………………………………………………….. 2

บทนำ …………………………………………………………………………….. 4

ความสำคัญและที่มาของปัญหา …………………………………………………. 4

การทบทวนวรรณกรรมที่เกี่ยวข้อง ……………………………………………… 5

คำถามของการวิจัย……………………………………………………………….. 5

วัตถุประสงค์ของการวิจัย ………………………………………………………… 6

สมมติฐานของการวิจัย……………………………………………………………. 6

ประโยชน์ที่คาดว่าจะได้รับจากการวิจัย …………………………………………. . 6

คำสำคัญ………………………………………………………………………..…. 7

การดำเนินการวิจัย ……………………………………………………………….. 7

- รูปแบบการวิจัย…………………………………………………………… 7
- นิยามตัวแปรที่เกี่ยวข้อง……………………………………………….…. 7
- ประชากรกลุ่มตัวอย่าง …………………………………………………… 8
- เกณฑ์ในการเลือกเข้ามาศึกษา………………………………………….... 8

**สารบัญเรื่อง(ต่อ)**

หน้า

- เกณฑ์ในการคัดออกจากการศึกษา ……………………………………….. 9
- ขนาดตัวอย่าง……………………………………………………………... 9
- การสุ่มตัวอย่าง............................................................................................. 10
- การสร้างซองจดหมายทึบ............................................................................. 11
- ตัวแปรในงานวิจัย………………………………………………………… 12
- อุปกรณ์ที่ใช้ในการวิจัย…………………………………………………… 12
- เครื่องมือที่ใช้วัดตัวแปร………………………………………………….. 12
- วิธีดำเนินการวิจัย…………………………………………………………. 13

การวิเคราะห์ข้อมูลและสถิติ ……………………………………………………... 15

ข้อพิจารณาด้านจริยธรรม……………………………………………………........ 17

ตารางเวลาการทำวิจัย…………………………………………………………......... 19

งบประมาณ……………………………………………….…………………......... 20

เอกสารอ้างอิง ………………………………………………………………..…... 21

ภาคผนวก ก หนังสือแสดงความยินยอมเข้าร่วมในการวิจัย……………………… 22

ภาคผนวก ข เอกสารชี้แจงผู้เข้าร่วมวิจัย……………………………………......... 25

ภาคผนวก ค แบบฟอร์มการเก็บข้อมูล………………………..………………….. 31

**บทนำ**

**ความสำคัญและที่มาของปัญหา**

ในประเทศไทยตั้งแต่อดีตมาหญิงหลังคลอดมีความสนใจเรื่องอาหารเพิ่มน้ำนม หรือหาซื้อยาแผนปัจจุบัน ยาสมุนไพรทั้งไทยและจีน มารับประทานเองเพื่อให้ปริมาณน้ำนมเพิ่มมากขึ้นให้เพียงพอต่อความต้องการของทารก การศึกษาในต่างประเทศรายงานว่ายา domperidoneสามารถใช้เป็นสารกระตุ้นน้ำนมได้ domperidoneเป็นสารกระตุ้นน้ำนมชนิดเดียวที่มี randomized controlled trial พบว่ามีประสิทธิภาพและมีความปลอดภัยในการใช้เพิ่มปริมาณน้ำนม^1^แต่เมื่อเร็วๆนี้องค์การอาหารและยาของสหรัฐอเมริกา (USFDA) มีคำเตือนเกี่ยวกับการใช้ยาชนิดนี้ที่ให้ทางหลอดเลือดดำ^2,3^ อย่างไรก็ตามยังไม่มีรายงานผลเสียต่อมารดาหลังคลอดและลูกที่ได้รับยานี้โดยการรับประทาน^4^การใช้ยาสมุนไพรไทยจึงเป็นอีกทางเลือกหนึ่ง

การใช้ยาสมุนไพรหลังคลอดมีวัตถุประสงค์หลายอย่าง เช่น เพื่อขับน้ำคาวปลา ขับเลือดเสีย ช่วยให้มดลูกเข้าอู่เร็ว บำรุงร่างกาย บำรุงน้ำนม ส่วนใหญ่จะมีฤทธิ์เผ็ดร้อน เนื่องจากในการแพทย์แผนไทยมีความเชื่อว่า ร่างกายของคนเราประกอบด้วยธาตุ 4 ได้แก่ ธาตุดิน ธาตุน้ำ ธาตุไฟ และธาตุลม โดยยึดหลักความสมดุลของธาตุทั้ง 4 เมื่อใดก็ตามที่เกิดความแปรปรวนจะมีความโน้มเอียงให้เกิดโรคหรืออาการที่ผิดปกติได้ ในภาวะหลังคลอดธาตุทั้ง 4 อยู่ในภาวะไม่สมดุล เนื่องจากการคลอดต้องใช้แรงเบ่ง ร่างกายสูญเสียน้ำ เสียเลือด อ่อนเพลีย ธาตุน้ำและธาตุลมในร่างกายเสียสมดุล ยังผลให้ธาตุไฟหย่อน การกินยารสร้อนจะช่วยให้ร่างกายอบอุ่น เป็นการบำรุงธาตุไฟ เมื่อธาตุไฟบริบูรณ์จะส่งผลให้ระบบต่างๆของร่างกายกลับคืนสู่สภาวะปกติได้เร็วขึ้น^5^

การใช้สมุนไพรในการกระตุ้นน้ำนมเป็นที่นิยมทั่วโลก^6^มีการใช้อย่างแพร่หลายตามค่านิยมและวัฒนธรรมท้องถิ่นมาเป็นเวลานานโดยไม่พบว่ามีอันตรายและมีประสิทธิภาพดี ทั้งที่ยังไม่ทราบกลไกการออกฤทธิ์แน่ชัดจนถึงปัจจุบันมีงานวิจัยเกี่ยวกับสมุนไพรไทยในการกระตุ้นน้ำนมน้อยมาก จากการศึกษาของ นพ.จักรกฤษณ์ สุรการ^7^เรื่องชาสมุนไพรบำรุงน้ำนม ที่มีส่วนผสมของฝาง ชะเอม มะตูม ขิง และเถาวัลย์เปรียง ในมารดาหลังคลอดโดยมีสรรพคุณบำรุงนํ้านม บำรุงร่างกาย และลดความปวดเมื่อยกล้ามเนื้อ มาใช้กับมารดาหลังคลอดเพื่อแก้ปัญหาทารกตัวเหลืองเนื่องจากได้รับปริมาณนํ้านมไม่เพียงพอพบว่า ยอดทารกตัวเหลืองลดลงจากร้อยละ 10 เหลือร้อยละ 3.06 นํ้านมมารดามาภายใน 24 ชั่วโมงร้อยละ 100

จากการติดตามเยี่ยมบ้านไม่พบปัญหาจากการใช้ชาชงสมุนไพรบำรุงนํ้านมทั้งต่อมารดาและทารก โดยมียอดมารดาที่ได้รับชาชงสมุนไพรบำรุงนํ้านมตั้งแต่เริ่มโครงการคือเดือนธันวาคม 2552 จนถึงเดือนสิงหาคม 2554 จำนวนทั้งสิ้น 1,311 คน แต่ยังไม่ได้มีการศึกษาในเชิงลึกและการเก็บข้อมูลอย่างเป็นระบบต่อมาทีมวิจัยรพ.วังน้ำเย็นได้ทำการทดลองโดยเปรียบเทียบชาสมุนไพรบำรุงน้ำนมกับชาใบเตยในการกระตุ้นน้ำนมมารดาหลังคลอดจำนวน 100 คน โดยกลุ่มทดลอง 50 คน และกลุ่มควบคุม 50 คน ตั้งแต่เดือนกันยายน 2554 ถึง เดือนพฤศจิกายน 2554 พบว่าชาสมุนไพรบำรุงน้ำนมได้ผลดีในการกระตุ้นน้ำนมมารดาหญิงหลังคลอดเมื่อเทียบกับชาใบเตยผู้ทำวิจัยจึงมีความสนใจที่จะทำการศึกษาต่อเนื่องจากงานวิจัยเดิมเพื่อเปรียบเทียบประสิทธิผลของชาสมุนไพรกับ Domperidoneซึ่งเป็น galactogueที่นิยมใช้กัน เพื่อเป็นทางเลือกในการดูแลมารดาหลังคลอดบุตรที่มีปัญหาน้ำนมไม่เพียงพอต่อไป โดยผู้วิจัยเลือกใช้ขนาดยา domperidone 30 mg/day เนื่องจากมีหลักฐานการใช้ยาในระดับสูงกว่านี้ไม่ได้เพิ่มปริมาณน้ำนม^8^แต่อาจเพิ่มความเสี่ยงต่อการเกิดผลข้างเคียงได้และเลือกกลุ่มประชากรที่จะศึกษาเฉพาะในมารดาหลังผ่าตัดคลอด อันเป็นหนึ่งในปัจจัยหลายประการที่ทำให้หญิงหลังคลอดมีปริมาณน้ำนมไม่เพียงพอต่อความต้องการของทารก^9^

**คำถามการวิจัย**

ชาสมุนไพรบำรุงน้ำนมรพ.วังน้ำเย็นที่มีส่วนผสมของฝาง ชะเอม มะตูม ขิงและเถาวัลย์เปรียง สามารถใช้เพิ่มปริมาณน้ำนมในมารดาหลังได้หรือไม่ และเมื่อเทียบกับยาเม็ดดอมเพอริโดนซึ่งเป็นสารกระตุ้นน้ำนมที่นิยมใช้กันแพร่หลายแล้ว อะไรมีประสิทธิภาพดีกว่ากัน

**วัตถุประสงค์ของการวิจัย**

วัตถุประสงค์หลัก

เพื่อศึกษาเปรียบเทียบประสิทธิผลของชาสมุนไพรบำรุงน้ำนม(รพ.วังน้ำเย็น) และdomperidoneต่อปริมาณน้ำนมของมารดา3 วันแรกหลังคลอด

วัตถุประสงค์รอง

1. เพื่อศึกษาเปรียบเทียบผลข้างเคียงของชาสมุนไพรบำรุงน้ำนม(รพ.วังน้ำเย็น)และ domperidone ต่อมารดาหลังคลอดบุตร เช่น ปากแห้ง ปวดศีรษะ ปวดท้องเป็นต้น
2. เพื่อศึกษาเปรียบเทียบผลของชาสมุนไพรบำรุงน้ำนม(รพ.วังน้ำเย็น)และ domperidone ต่อภาวะแทรกซ้อนของทารก เช่นตัวเหลือง เป็นต้น
3. เพื่อศึกษาเปรียบเทียบผลของชาสมุนไพรบำรุงน้ำนม(รพ.วังน้ำเย็น)และ domperidone ต่อภาวะแทรกซ้อนของมารดาหลังคลอด เช่น การตกเลือดหลังคลอด การติดเชื้อหลังคลอด เป็นต้น
4. เพื่อศึกษาความพึงพอใจของมารดาหลังคลอดที่ได้รับชาสมุนไพรบำรุงน้ำนมและ domperidone

**สมมติฐานการวิจัย**

H0 = ชาสมุนไพรบำรุงน้ำนม(รพ.วังน้ำเย็น) และ domperidone ไม่สามารถเพิ่มปริมาณน้ำนมในมารดาหลังผ่าตัดคลอดบุตรได้

Ha = ชาสมุนไพรบำรุงน้ำนม(รพ.วังน้ำเย็น) และ domperidone สามารถเพิ่มปริมาณน้ำนมในมารดาหลังผ่าตัดคลอดบุตรได้

**ประโยชน์ที่คาดว่าจะได้รับ**

1.นำไปสู่การดูแลหญิงตั้งครรภ์ที่มีปัญหาเรื่องน้ำนมไม่เพียงพอ เพื่อเพิ่มปริมาณน้ำนมให้เพียงพอต่อความต้องการของทารก

2.เป็นแนวปฏิบัติสำหรับเจ้าหน้าที่สาธารณสุขในการนำสมุนไพรของไทยซึ่งหาได้ง่าย ผลิตเองได้ ราคาถูก มาใช้ในการส่งเสริมการเลี้ยงลูกด้วยนมแม่ให้ประสบผลสำเร็จ

3.เป็นแนวทางของผู้บริหารและหน่วยงานที่เกี่ยวข้องในการนำข้อมูลและความรู้ที่ได้จากการวิจัยนำมาปรับปรุง กำหนดนโยบายตลอดจนวางแผนการดำเนินงานส่งเสริมการเลี้ยงลูกด้วยนมแม่และพัฒนาการแพทย์แผนไทยให้ประสบผลสำเร็จ

**คำสำคัญ (key words)**

Wang Nam Yen Herbal Tea, Domperidone, Human milk production, postpartum

**การดำเนินการวิจัย**

**รูปแบบการวิจัย**

การศึกษาแบบทดลองโดยมีการสุ่มกลุ่มตัวอย่าง (Randomized double blind ,placebo controlled trial)

**นิยามตัวแปรที่เกี่ยวข้อง**

- Domperidone หมายถึง สารกระตุ้นน้ำนมที่มีกลไกยับยั้ง dopamine ใช้ในการรักษากรดไหลย้อนและอาการคลื่นไส้อาเจียน ยา domperidone ทำให้ระดับ prolactin ในเลือดสูงขึ้นแม้จะใช้ในหญิงที่ไม่ตั้งครรภ์เป็นสารกระตุ้นน้ำนมชนิดเดียวที่มี randomized controlled trial พบว่ามีประสิทธิภาพและมีความปลอดภัยในการใช้เพิ่มปริมาณน้ำนม ผลข้างเคียงที่พบได้บ่อยคือ ปากแห้ง ปวดศีรษะ (อาการมักจะดีขึ้นเมื่อลดขนาดยา) และอาการปวดเกร็งท้องการใช้ยาในขนาดสูงเป็นเวลานานในหนูทดลองพบว่ามีอุบัติการณ์ของเนื้องอกเต้านมมากขึ้นแต่ไม่พบรายงานในมนุษย์ domperidone มีข้อห้ามใช้ใน คนที่แพ้ยา และในภาวะที่การกระตุ้นลำไส้อาจทำให้เกิดอันตรายเช่น เลือดออกในทางเดินอาหาร ลำไส้อุดตัน หรือลำไส้ทะลุ องค์การอาหารและยาของสหรัฐอเมริกา (USFDA)มีคำเตือนเกี่ยวกับการใช้ยาชนิดที่ให้ทางหลอดเลือดดำ แต่ไม่พบว่าการใช้ยาชนิดรับประทานมีผลเสียต่อแม่และลูก ขนาดยาที่ใช้ในการวิจัยนี้คือ 10มก. 3ครั้งต่อวัน นาน 3วัน Domperidoneที่ใช้ในการทดลอง มีชื่อทางการค้าว่า Mirax-M ยี่ห้อ Berlin
- การผ่าท้องทำคลอด (Cesarean section) หมายถึง การผ่าตัดเพื่อคลอดทารกผ่านทางหน้าท้อง ในช่วงอายุครรภ์ที่ทารกสามารถมีชีวิตรอดได้ ซึ่งโดยปกติแล้วแพทย์จะผ่าท้องทำคลอดก็ต่อเมื่อมีเหตุผลทางการแพทย์ โดยพิจารณาในรายที่ไม่สามารถคลอดทางช่องคลอดได้เอง หรือคลอดได้แต่อาจก่อให้เกิดอันตรายต่อมารดาหรือทารก
- ชาสมุนไพรบำรุงน้ำนม หมายถึง ชาชงสมุนไพรที่ประกอบด้วย ฝาง ชะเอม มะตูม ขิง เถาวัลย์เปรียง ซึ่งมีสรรพคุณ บำรุงน้ำนม บำรุงร่างกาย ลดอาการปวดเมื่อยกล้ามเนื้อภายหลังการคลอดบุตร^7^ ยานี้ได้รับการผลิตและตรวจสอบคุณภาพตามมาตรฐานการผลิตยาสมุนไพรจากโรงพยาบาลวังน้ำเย็นวิธีรับประทาน ใช้ชา จำนวน 1 ซองชงในน้ำอุ่น 200 มิลลิลิตรนาน 5นาที รับประทานหลังอาหาร 30 นาทีเช้า กลางวันและเย็น เป็นเวลา 3 วันจากการศึกษาของจักรกฤษณ์ สุรการและคณะ ไม่พบอาการไม่พึงประสงค์ในกลุ่มที่ใช้ชาสมุนไพรบำรุงน้ำนมชนิดนี้
- ปริมาณน้ำนม หมายถึง ปริมาณน้ำนมของมารดาที่วัดได้ด้วยเครื่องปั้มนมไฟฟ้า หลังผ่าตัดคลอดทุก 24 ชั่วโมง จนครบ 72 ชั่วโมง โดยเริ่มปั๊มนมหลังจากบุตรดูดนมจนเกลี้ยงเต้า 2 ชั่วโมง นาน 15 นาที
- ชาหลอก หมายถึงเครื่องดื่มที่ปรุงแต่งให้มีลักษณะคล้ายชาสมุนไพรบำรุงน้ำนมที่ใช้ในการทดลอง (ใช้ใบเตยเป็นวัตถุดิบ)
- ยาหลอก หมายถึง เม็ดแป้งที่ถูกดัดแปลงให้มีลักษณะคล้าย Domperidoneที่ใช้ในการทดลอง (ใช้แป้งมันสำปะหลังเป็นวัตถุดิบ)
- ปริมาณน้ำนมไม่เพียงพอ หมายถึง สตรีตั้งครรภ์หลังคลอดบุตรไม่สามารถผลิตปริมาณน้ำนมได้เพียงพอต่อความต้องการของทารกในแต่ละวัน

**ประชากร**

สตรีตั้งครรภ์ที่คลอดบุตรที่มีปัญหาปริมาณน้ำนมไม่เพียงพอต่อความต้องการของทารก

**กลุ่มตัวอย่าง**

สตรีตั้งครรภ์ที่คลอดบุตรโดยการผ่าตัดคลอดที่โรงพยาบาลสรรพสิทธิประสงค์ อายุ 15 ปีถึง 41 ปี อายุครรภ์ 28-42 สัปดาห์

**เกณฑ์การเลือกเข้ามาศึกษา (Inclusion criteria)**

สตรีตั้งครรภ์ที่คลอดบุตรอายุ 15 ปีถึง 41 ปี อายุครรภ์ 28-42 สัปดาห์ ที่ได้รับการผ่าตัดคลอดบุตรที่โรงพยาบาลสรรพสิทธิประสงค์ ทุกรายที่ยินยอมเข้าร่วมการวิจัย

**เกณฑ์ในการคัดออกจากการศึกษา (Exclusion criteria)**

1. มีข้อห้ามในการให้นมบุตรเช่นติดเชื้อ HIV
2. มีภาวะแทรกซ้อนรุนแรงที่เกิดขึ้นระหว่างการคลอด ทำให้ไม่สามารถเลี้ยงนมบุตรได้ เช่น severe postpartum hemorrhage with hypovolemic shock, cardiac arrest, severe preeclampsia และ eclampsia เป็นต้น
3. มีประวัติแพ้ยา domperidone หรือ ส่วนผสมของชาสมุนไพรกระตุ้นน้ำนม
4. มารดาที่มีบุตรป่วยได้แยกจากมารดาตั้งแต่หลังคลอด เพื่ออยู่ในความดูแลของกุมารแพทย์อย่างใกล้ชิด ไม่สามารถให้ทารกดูดนมจากเต้านมได้

**ขนาดตัวอย่าง**

คำนวณขนาดตัวอย่างจาก สูตรการเปรียบเทียบความแตกต่างของค่าเฉลี่ยในประชากร 2กลุ่ม

โดยอ้างอิงจากงานวิจัยของ Surasak Jantarasaeng^10^ซึ่งเปรียบเทียบ domperidoneกับยาหลอก ภายใต้สมมติฐานว่าชาสมุนไพรบำรุงน้ำนมจะสามารถเพิ่มปริมาณน้ำนมได้ไม่ด้อยกว่า domperidone

พบว่า Mean daily milk volume in the domperidone group day 4= 191.3(SD=136.1)

Mean daily milk volume in the placebo group day4 = 91.4 (SD=60.3)

n/group = $\frac{3{(Z\alpha+Z\beta)}^{2}\delta^{2}}{\left( \mu_{1}-\mu_{2} \right)^{2}}$

โดย $\delta=SDของกลุ่มผลลัพธ์หลัก$

$$\mu_{1}=191.3 (case group)$$

$$\mu_{2} =91.4 (control group)$$

กำหนดให้α = 0.05, Zα = 1.65 อำนาจการทดสอบ 80% Zβ = 0.84

n/group = $\frac{3\left( 1.65+0.84 \right)^{2}{(136.1)}^{2}}{{(191.3-91.4)}^{2}}$

ได้ n/group = 34.52

เมื่อพิจารณาว่าอาจจะมีการสูญเสียกลุ่มตัวอย่าง 15% จึงได้กลุ่มตัวอย่างกลุ่มละ 40 คน

แบ่งเป็น 3กลุ่ม ดังนี้

1.กลุ่มที่ได้รับชาสมุนไพรบำรุงน้ำนมและยาหลอก40 คน

2.กลุ่มที่ได้รับdomeridoneและชาหลอก 40คน

3.กลุ่มที่ได้รับชาหลอกและยาหลอก 40 คน

**การสุ่มตัวอย่าง**

อาสาสมัครที่เข้าร่วมโครงการจะถูกแบ่งออกเป็น 3 กลุ่ม แบบสุ่มในอัตราส่วน 1 : 1 : 1 โดยใช้วิธี Block of three ร่วมกับตารางเลขสุ่มเพื่อสร้างรหัสสุ่ม หลังจากการได้รหัสสุ่มแล้ว รหัสสุ่มจะถูกเก็บไว้ในซองทึบและมีการปกปิดไม่ให้ผู้ให้คำปรึกษา(clinician) และผู้เก็บข้อมูล(Data collector) ทราบว่าอาสาสมัครอยู่ในกลุ่มใด

- **การสร้างรหัสสุ่ม**

ขั้นตอนการสร้างรหัสสุ่ม จะกำหนดให้

A = กลุ่มT คือ กลุ่มที่ได้รับชาสมุนไพรบำรุงน้ำนม(รพ.วังน้ำเย็น) และยาหลอกของดอมเพอริโดน

B = กลุ่มD คือกลุ่มที่ได้รับยาเม็ดดอมเพอริโดนและชาใบเตย(ชาหลอก)

C = กลุ่มC คือกลุ่มที่ได้รับยาหลอกของดอมเพอริโดนและชาใบเตย(ชาหลอก)

เมื่อใช้ Block of three จะสามารถเรียงลำดับความน่าจะเป็นได้ 6 แบบ คือ 1 = ABC, 2 = ACB, 3 = BAC, 4 = BCA, 5 = CAB และ 6 = CBA

จากนั้นนำหมายเลขที่ได้ไปเข้าตารางเลขสุ่มโดยหมายเลข 0, 7, 8 และ 9 ไม่ใช้

จากตารางเลขสุ่ม เริ่มจากแถวที่ 2 สดมภ์ที่ 3 ไปทางขวามือจะได้ตัวเลขเรียงลำดับดังนี้

เมื่อได้รหัสสุ่มครบ 120 รหัสสุ่มแล้ว นำรหัสสุ่มที่ได้จากตารางเลขสุ่มมาเรียงต่อกันโดยเริ่มจากตำแหน่งที่ 9 (TD009) เป็นลำดับแรก และเรียงต่อกันจนครบ 120 รหัสสุ่ม

- **การสร้างซองจดหมายทึบ**

หลังจากได้รหัสแบ่งกลุ่มชาสมุนไพรบำรุงน้ำนม(รพ.วังน้ำเย็น) กลุ่ม domperidone และกลุ่มControl แล้ว ทีมผู้วิจัยที่รับผิดชอบในการผลิตซองจดหมาย จะทำการผลิตซองจดหมายทึบปิดผนึก โดยหน้าซองจะมีรายละเอียด ได้แก่ ชื่อโครงการวิจัย, ชื่อผู้วิจัยหลัก, สถานที่ดำเนินการ, รหัสการสุ่ม (Randomization ID), รหัสอาสาสมัคร (Study ID) และวันที่เปิดซองจดหมาย ดังแสดงในรูป

Study : Efficacy of Wang Nam Yen Herbal Tea on Breast Milk Production : A Factorial Randomized Controlled Trial (Tea4Milk)

PI : Koollachart Saejueng

Site : Sunpasitthiprasong hospital

Randomization ID : TD001

Study ID : ________________________________________

Date envelope opened : ____________________________

ตัวอย่างหน้าซองจดหมายทึบที่ใช้แบ่งกลุ่มอาสาสมัครแบบสุ่ม

ในซองจดหมายจะมีรายละเอียดได้แก่ รหัสการสุ่ม(Randomization ID), กลุ่มที่อาสาสมัครได้รับเลือก, รหัสอาสาสมัคร(Study ID), วันที่เปิดซองจดหมาย, เวลาที่เปิดซองจดหมาย, ลายเซ็นของผู้ที่เปิดซองและทำหน้าที่ลงทะเบียนสุ่มแบ่งกลุ่มอาสาสมัคร ดังแสดงในรูป

Randomization ID : TD001

Assessment group :

Study ID : ________________________________________

Date envelope opened : ____________________________

Time envelope opened : ____________________________

Signature the person who randomizing the subject :

________________________________________________

ตัวอย่างรายละเอียดในซองจดหมายทึบที่ใช้แบ่งกลุ่มอาสาสมัครแบบสุ่ม

อาสาสมัครที่ผ่านการคัดกรองและลงนามยินยอมเข้าร่วมโครงการวิจัยแล้ว จะได้รหัสอาสาสมัคร (Study ID) และให้คำปรึกษารายบุคคล และหยิบซองจดหมายเพื่อแบ่งกลุ่มอาสาสมัคร ที่หน้าซองจดหมายจะใส่รหัสอาสาสมัครและเวลาที่อาสาสมัครหยิบซองในช่องที่ว่างไว้ และถูกส่งไปที่ศูนย์วิจัยหลัก เพื่อให้ผู้ที่ทำหน้าที่ลงทะเบียนแบ่งกลุ่มอาสาสมัครเปิดซอง และเขียนรหัสอาสาสมัคร, วันที่เปิดซอง, เวลาที่เปิดซอง และลายเซ็นของผู้เปิดซองและลงทะเบียน ซึ่งผู้เปิดซองจะนำข้อมูลไปลงในทะเบียน Randomization log book และซองจดหมายที่ได้รับการเปิดซองจะถูกปิดผนึกอีกครั้ง และเก็บรักษาไว้เป็นความลับ

ซองจดหมายที่ถูกเก็บไว้เป็นความลับ จะถูกนำมาเปิดใหม่อีกครั้งเพียงกรณีเดียวคือ อาสาสมัครเกิดภาวะแทรกซ้อนที่อันตรายและรุนแรง ซึ่งน่าสงสัยว่าเกิดจากผลของกิจกรรมในกลุ่มทดลอง (Intervention group) ผู้ที่ทำหน้าที่เก็บทะเบียน Randomization log book จะเก็บไว้เป็นความลับโดยปกปิดไม่ให้ผู้ให้คำปรึกษา (Clinician), ผู้เก็บข้อมูล (Data collector) ทราบว่าอาสาสมัครอยู่ในกลุ่มใด

**ตัวแปรในงานวิจัย**

ตัวแปรตาม = ปริมาณน้ำนมที่วัดได้ในวันที่ 3 หลังคลอด

ตัวแปรอิสระ = การได้รับยา domperidone หรือ ชาสมุนไพรบำรุงน้ำนม(รพ.วังน้ำเย็น)

**อุปกรณ์ที่ใช้ในการวิจัย**

1. Electronic breast pump (Spectra2^R^)
2. แบบบันทึกข้อมูลผู้เข้าร่วมโครงการวิจัย( case record form)

**เครื่องมือที่ใช้เก็บข้อมูล**

คือ แบบบันทึกข้อมูลการศึกษาเปรียบเทียบประสิทธิผลของ domperidone และชาสมุนไพรบำรุงน้ำนม(รพ.วังน้ำเย็น) ซึ่งผู้วิจัยจัดทำขึ้นและทดสอบแล้ว เพื่อจดบันทึกข้อมูลจากระเบียนประวัติของมารดาและทารก ปริมาณน้ำนมที่วัดได้ ผลการคลอดและภาวะแทรกซ้อนต่างๆ ในสตรีตั้งครรภ์ที่มารับการผ่าตัดคลอด อายุครรภ์ 28^+0^ สัปดาห์ ถึง 42^+0^ สัปดาห์ทุกรายที่ยินยอมเข้าร่วมการวิจัยในช่วงเดือนกุมภาพันธ์2560 – เดือนกันยายน 2560 จำนวน 120 คน

**วิธีดำเนินการวิจัย**

1. ศึกษาข้อมูลและทบทวนวรรณกรรม
2. เขียนโครงร่างงานวิจัยและออกแบบบันทึกข้อมูลเสนอต่อคณะกรรมการจริยธรรมเพื่อขออนุญาตทำงานวิจัย
3. สตรีตั้งครรภ์ที่คลอดบุตร อายุครรภ์ 28-42 สัปดาห์ ที่ได้รับการผ่าตัดคลอดบุตรที่โรงพยาบาลสรรพสิทธิประสงค์ ที่ยินยอมเข้าร่วมในงานวิจัยจะได้รับการสอนเกี่ยวกับเรื่องการให้นมบุตรตามมาตรฐานการดูแลหญิงหลังคลอด
4. ขอความร่วมมือและอธิบายรายละเอียดรวมถึงรูปแบบของโครงการวิจัยแก่อาจารย์แพทย์ แพทย์ประจำบ้าน พยาบาลห้องคลอด และพยาบาลหอผู้ป่วยสูติกรรม ในการคัดเลือกหญิงตั้งครรภ์ที่เข้าเกณฑ์คัดเลือกเข้าร่วมการวิจัยและเกณฑ์การคัดเลือกออกจากการวิจัย
5. ให้คำแนะนำและอธิบายเกี่ยวกับรายละเอียดของโครงการให้แก่ผู้ที่ถูกคัดเลือกให้เข้าร่วม

โครงการวิจัยเพื่อชักชวนให้เข้าร่วมโครงการ

แนวทางหลักคือ แพทย์ผู้รักษาและพยาบาลประจำหอผู้ป่วยหลังคลอดจะเป็นผู้อธิบายถึงรายละเอียดโครงการวิจัย และชักชวนให้สตรีตั้งครรภ์เข้าร่วมโครงการ โดยเป็นสตรีหลังผ่าตัดคลอดบุตร และเข้าได้ตามเกณฑ์การเข้าร่วมโครงการ

แนวทางปฏิบัติการขอความยินยอมเข้าร่วมโครงการวิจัย (Informed consent process) เจ้าหน้าที่วิจัย (แพทย์และพยาบาลวิจัย) จะอธิบายให้ข้อมูลแก่อาสาสมัครอย่างสมบูรณ์ในทุกด้านของการวิจัยโดยใช้เอกสารแนะนำสำหรับอาสาสมัครฉบับภาษาไทยซึ่งต้องได้รับการรับรองจากคณะกรรมการจริยธรรมวิจัยในมนุษย์ของหน่วยงาน เจ้าหน้าที่วิจัยจะต้องทำให้มั่นใจว่าก่อนที่อาสาสมัครจะลงนามยินยอมเข้าร่วมโครงการวิจัยนี้ อาสาสมัครเข้าใจอย่างดีเกี่ยวกับ วัตถุประสงค์ของการศึกษาวิจัย กระบวนการวิจัย และผลประโยชน์หรือความเสี่ยงใดที่อาจขึ้นขณะเข้าร่วมงานวิจัย อาสาสมัครจะมีเวลามากเพียงพอที่จะศึกษาเอกสารแนะนำและใบยินยอมด้วยความสมัครใจ (Informed consent form) และจะมีโอกาสที่จะซักถามประเด็นหรือข้อสงสัยเกี่ยวกับการวิจัยนี้ อาสาสมัครจะต้องเข้าใจอย่างดีว่าการเข้าร่วมการวิจัยนั้นขึ้นกับการตัดสินใจของอาสาสมัครเอง โดยอาสาสมัครอาจตัดสินใจไม่เข้าร่วมการวิจัยตั้งแต่ต้น หรือสามารถหยุดหรือถอนตัวจากการเข้าร่วมการวิจัยได้ตลอดเวลาของการศึกษาโดยที่การตัดสินใจไม่เข้าร่วมการศึกษาวิจัยดังกล่าวจะไม่ทำให้เกิดความแตกต่างในการดูแลรักษาทั้งในปัจจุบันและในอนาคต สำเนาของใบยินยอมด้วยความสมัครใจ 1 ชุดจะให้ไว้กับอาสาสมัครเพื่อเก็บไว้ กระบวนการในการขอความยินยอมในการเข้าร่วมโครงการวิจัยนี้จะดำเนินการในห้องส่วนตัวที่เป็นสัดส่วน จะไม่มีกระบวนหรือกิจกรรมการวิจัยใดเกิดขึ้นก่อนอาสาสมัครจะลงนามยินยอมเข้าร่วมโครงการวิจัย

1. อาสาสมัครที่เข้าร่วมโครงการจะถูกออกเป็น 3 กลุ่ม แบบสุ่มโดยใช้ block of threeและตารางเลขสุ่มได้กลุ่มละ 40คนรวมกลุ่มตัวอย่างทั้งหมดได้ 120คน ดังนี้

3.1)กลุ่มที่ได้รับชาสมุนไพรบำรุงน้ำนมและยาหลอก 40 คน(T)

3.2)กลุ่มที่ได้รับdomeridoneและชาหลอก 40คน(D)

3.3)กลุ่มที่ได้รับชาหลอกและยาหลอก 40 คน (C)

1. Intervention ที่สตรีตั้งครรภ์จะได้รับ จะถูกบรรจุเป็นห่ออย่างดีจากแหล่งผลิต โดยเภสัชกรและ พยาบาลที่ทำหน้าที่จ่ายยาจะไม่ทราบว่าเป็น intervention ใด จะมีรหัสตั้งแต่ TD001ไปจนถึง TD120 โดยจ่ายเรียงตามลำดับที่อยู่บนบรรจุภัณฑ์ให้แก่สตรีตั้งครรภ์ที่เข้าสู่การทดลอง เริ่มให้interventionที่12-18 ชั่วโมงหลังผ่าตัดคลอด
2. ดำเนินการวิจัย เริ่มการวิจัยโดย การให้intervention ตามชนิดของกลุ่มที่อาสาสมัครสุ่มได้ ตั้งแต่เริ่มเข้าร่วมงานวิจัย

- กลุ่มที่ได้Domperidone (10 mg) และ placebo ของ domperidone : รับประทาน1 เม็ด หลังอาหาร เช้า กลางวัน และเย็น เป็นเวลา 3วัน
- กลุ่มที่ได้ ชาชงสมุนไพรและ placebo ของชาชงสมุนไพร : ใช้ชาชงสมุนไพร 1 ซองชา( 1 ซองมี 10 ซองชา) แช่น้ำอุ่น 200 cc นาน 5 นาที ดื่มหลังอาหาร 30 นาที เช้า กลางวัน และเย็น เป็นเวลา 3วัน

1. บันทึกข้อมูลพื้นฐานของมารดา เช่น อายุ อาชีพ รายได้ สิทธิการรักษา ระดับการศึกษา ลักษณะการเลี้ยงดูบุตรประสบการณ์การเลี้ยงบุตรด้วยน้ำนมในท้องก่อน จำนวนครั้งที่ตั้งครรภ์ จำนวนการคลอดบุตร อายุครรภ์ โรคประจำตัว ประวัติการผ่าตัด ภาวะแทรกซ้อนระหว่างการตั้งครรภ์ ข้อบ่งชี้การผ่าตัดคลอด ผลการคลอดน้ำหนักทารก Apgar ปัญหาของทารกแรกเกิด เช่นตัวเหลือง การนอนรักษาตัวในหอทารกวิกฤติ ภาวะแทรกซ้อนหลังคลอดของมารดา เช่น ภาวะตกเลือดหลังคลอด การติดเชื้อหลังคลอด
2. ให้มารดาเก็บน้ำนมหลังจากได้ intervention24-72ชั่วโมง โดยใช้เครื่องปั๊มนมอัตโนมัติ ยี่ห้อ spectraรุ่น spectra 2 ซึ่งมีแรงดูดต่ำสุด 10 mmHg แรงดูดสูงสุด 300 mmHg วงจรการดูดประมาณ 42 ครั้งต่อนาทีวัดปริมาณเป็นมิลลิลิตร กำหนดเวลาการปั๊มนมหลังจากลูกดูดนมครั้งสุดท้าย 2 ชม.ปั๊มนานข้างละ 15 นาที บันทึกปริมาณน้ำนมที่เก็บได้
3. บันทึกผลข้างเคียงระหว่างการให้ยา ได้แก่ ปากแห้ง ปวดหัว นอนไม่หลับ ปวดท้อง ท้องเสีย คลื่นไส้และปัสสาวะไม่ออก
4. บันทึกปริมาณน้ำที่ดื่มระหว่างวัน และปริมาณปัสสาวะต่อวัน จำนวนครั้งที่ให้นมบุตรต่อวัน
5. บันทึกน้ำหนักที่เปลี่ยนแปลงของทารกและมารดาหลังคลอด
6. ก่อนจำหน่ายมารดากลับบ้านวัด vital signs, ความสูงของยอดมดลูก สีน้ำคาวปลาสอบถามภาวะแทรกซ้อนทางมารดา เช่น ตกเลือดหรือติดเชื้อหลังคลอด สอบถามภาวะแทรกซ้อนของทารก เช่น ตัวเหลืองหรือหายใจผิดปกติ สอบถามการใช้ยาอื่นเสริม หรือกินอาหารเสริมเพื่อเพิ่มปริมาณน้ำนม สอบถามความพึงพอใจเกี่ยวกับการให้นมบุตรและการใช้ยากระตุ้นน้ำนม
7. ประมวลผลจากข้อมูลที่เก็บรวบรวมได้
8. รายงานผลการวิจัยในรูปแบบที่เหมาะสม
9. สรุปผลการวิจัย วิจารณ์ผล และส่งตีพิมพ์

**การวิเคราะห์ข้อมูลและสถิติ**

ใช้สถิติเชิงพรรณนาเบื้องต้นในการวิเคราะห์ข้อมูลเพื่ออธิบายลักษณะของประชากร/กลุ่มตัวอย่างที่ต้องการศึกษา เช่น จำนวน(ร้อยละ), ค่าเฉลี่ย(mean) (ส่วนเบี่ยงเบนมาตรฐาน (standard deviation: SD)), ค่ามัธยฐาน(Median) (interquatile range) โดยใช้สถิติ chi-square สำหรับข้อมูลเชิงคุณภาพ (categorical data) ใช้ histogram และ Shapiro–Wilk test เพื่อดูการกระจายข้อมูลเชิงปริมาณ โดยใช้ one-way ANOVA with Bonferroni correction และ Kruskall-Wallis test ข้อมูลเชิงปริมาณที่มีการกระจายตัวปกติ และข้อมูลเชิงปริมาณที่การกระจายตัวไม่ปกติ ตามลำดับ กำหนดค่าความเชื่อมั่นทางสถิติที่ *p*<0.05 ถือว่ามีความแตกต่างกันอย่างมีนัยสำคัญ ทำการวิเคราะห์ข้อมูลแบบ intention-to-treat โดยใช้โปรแกรม Stata/MP software version 15.0 (StataCorp 2017, College Station, TX).

**แผนภาพแสดงขั้นตอนการวิจัย**

ประชาสัมพันธ์โครงการวิจัยแก่มารดาหลังผ่าตัดคลอด
(ที่ตึกผู้ป่วยสูติกรรม 1,สูติกรรม 2,สูติกรรม 3 และพระปทุม 4)

เข้า inclusion criteria

มารดาเซ็นยินยอมเข้าร่วมโครงการวิจัย

แบ่งกลุ่มแบบ Randomization 3กลุ่ม

| T (40 คน)  ได้รับชาสมุนไพรบำรุงน้ำนมและยาหลอก | D (40 คน)  ได้รับ domperidoneและชาหลอก | C (40 คน)  ได้รับชาหลอกและยาหลอก |
| --- | --- | --- |

พยาบาลบันทึกข้อมูลทั่วไปลงในแบบฟอร์ม และเริ่มให้ intervention ที่ 12-18 ชั่วโมงหลังคลอด

พยาบาลบันทึก I/O , จำนวนครั้งที่ให้นมบุตรต่อวัน, ปริมาณน้ำนมเฉลี่ยต่อครั้งต่อเต้า และอาการข้างเคียงที่ 24ชั่วโมง, 48 ชั่วโมง,72 ชั่วโมง ตามลำดับ

ที่ 72ชั่วโมง ชั่งน้ำหนักมารดาและทารกและประเมิน

1.ปริมาณน้ำนม

2.อาการหลังคลอด ได้แก่ ยอดมดลูก, สีของน้ำคาวปลา, ภาวะแทรกซ้อนของมารดา เช่น ตกเลือด,ติดเชื้อ, ภาวะแทรกซ้อนของทารก เช่น ตัวเหลือง, หายใจผิดปกติ

3.ความพึงพอใจในการใช้ยากระตุ้นน้ำนมจำหน่ายผู้ป่วยกลับบ้านถ้าไม่มีภาวะแทรกซ้อน

**ข้อพิจารณาด้านจริยธรรม**

**ผลกระทบที่อาจเกิดแก่ผู้เข้าร่วมการวิจัยและการชดเชย**

**ความเสี่ยงด้านจิตใจ**

-อาสาสมัครอาจรู้สึกอึดอัดใจ รู้สึกไม่สะดวกสบาย เนื่องจากต้องกินยาเพิ่มทุกวัน เช้า กลางวัน เย็น และในกลุ่มที่ได้รับชาสมุนไพรอาจมีปัญหาเรื่องการรับประทานเนื่องจากรสชาติและลักษณะทางกายภาพของยาได้

-ผู้เข้าร่วมวิจัยต้องเข้ารับการปั้มเต้านมด้วยเครื่องปั้มนม ทำให้อาสาสมัครรู้สึกอึดอัดที่ต้องมาทำการปั้มเต้านม อาจเจ็บเต้านมขณะทำการปั้มได้ แต่อย่างไรก็ตาม ในขณะปั้มเต้านม จะทำในห้องปิดมิดชิด และทำการตรวจวัดโดยจ้าหน้าที่ผู้ชำนาญการและมีประสบการณ์ในการใช้เครื่องปั้มนม

**ความเสี่ยงด้านร่างกาย**

-อาจเกิดอาการไม่พึงประสงค์จากการใช้ยาได้

-การปั้มนมด้วยเครื่องปั้มนม อาจะทำให้อาสาสมัครเจ็บเต้านมเวลาปั้มนมได้ แต่ทางผู้วิจัยจะทำการปั้มนมอย่างนุ่มนวล ระมัดระวัง และทำการตรวจวัดโดยเจ้าหน้าที่ผู้ชำนาญการและมีประสบการณ์ในการใช้เครื่องปั้มนม

**ความเสี่ยงต่อการสูญเสียเวลา และโอกาสทางเศรษฐกิจและสังคม**

-อาจเป็นไปได้ที่การเข้าร่วมโครงการวิจัย จะทำให้อาสาสมัครสูญเสียเวลา และโอกาสทางเศรษฐกิจและสังคม เนื่องจาก ต้องมาตรวจเฝ้าติดตามและประเมินประสิทธิผลของ intervention ที่ได้รับ แต่การใช้เวลาในการเข้าร่วมการวิจัยดังกล่าวจะได้รับการชี้แจงอย่างชัดเจนก่อนที่อาสาสมัครจะตัดสินใจเข้าร่วมโครงการ และอาสาสมัครมีสิทธิที่จะปฏิเสธที่จะเข้าร่วมโครงการวิจัยตั้งแต่ต้น หรือ ณ เวลาใดขณะที่การศึกษาวิจัยกำลังดำเนินการอยู่ก็ได้

**ผู้วิจัยวางแผนที่จะป้องกันผลแทรกซ้อนและการดูแลรักษากรณีเกิดผลแทรกซ้อนอย่างไร**

**ด้านจิตใจ**

-การปั้มเต้านมด้วยเครื่องปั้มนม ทางผู้วิจัยจะทำการตรวจวัดอย่างนุ่มนวล ระมัดระวัง และทำการตรวจวัดโดยเจ้าหน้าที่ผู้ชำนาญการและมีประสบการณ์ในการใช้เครื่องปั้มนม ขณะทำการปั้มนมจะทำในห้องปิดมิดชิดไม่มีบุคคลอื่นมารบกวน

**ด้านร่างกาย**

-หากเกิดอาการไม่พึงประสงค์ ผู้วิจัยจะให้อาสาสมัครหยุดใช้ยาทันที และรักษาภาวะแทรกซ้อนอย่างรวดเร็ว เพื่อไม่ให้เกิดภาวะแทรกซ้อนรุนแรง และให้ผู้วิจัยออกจากการวิจัย

**การสูญเสียโอกาสทางเศรษฐกิจและสังคม**

-ผู้วิจัยจะได้กำหนดแนวทางให้การเข้ารับการตรวจประเมินประสิทธิผลของ intervention ใช้เวลาสั้นที่สุด โดยทำให้เสร็จสิ้นภายในสามวันขณะที่อาสาสมัครนอนพักรักษาตัวภายในโรงพยาบาล

**ใครเป็นผู้รับผิดชอบค่าใช้จ่ายในการรักษาพยาบาลกรณีเกิดผลแทรกซ้อน**

-ผู้วิจัยคาดว่าไม่น่าจะมีภาวะแทรกซ้อนจากการศึกษาวิจัยนี้ แต่หากเกิดกรณีภาวะแทรกซ้อนใดๆ ที่หลีกเลี่ยงไม่ได้ ทางผู้วิจัยและโรงพยาบาลจะเป็นผู้รับผิดชอบค่าใช้จ่ายในการรักษาพยาบาล ตามแนวทางมาตรฐานตามเวชปฏิบัติปกติของกระทรวงสาธารณสุข

**ตารางเวลาทำการวิจัย**

| **ขั้นตอนการทำงาน** | **มิ.ย..59** | **ก.ค.59** | **ส.ค.59** | **ก.ย.59** | **ต.ค.59** | **พ.ย.59** | **ธ.ค.59** | **ม.ค.60** | **ก.พ.60 ถึง ก.ย. 60** | | **ต.ค.60** | **พ.ย60** | **ธ.ค.60** |
| --- | --- | --- | --- | --- | --- | --- | --- | --- | --- | --- | --- | --- | --- |
| ศึกษาข้อมูลและทบทวนวรรณกรรม |  |  |  |  |  |  |  |  |  |  |  |  |  |
| เขียนโครงร่างการวิจัย |  |  |  |  |  |  |  |  |  |  |  |  |  |
| เสนอโครงร่างการวิจัยต่อภาควิชาสูติศาสตร์-นรีเวชวิทยา |  |  |  |  |  |  |  |  |  |  |  |  |  |
| เสนอโครงร่างการวิจัยและขอคำรับรองจากคณะกรรมการสิทธิ-มนุษยชนเกี่ยวกับการวิจัยในคน |  |  |  |  |  |  |  |  |  |  |  |  |  |
| ชี้แจงรายละเอียดเกี่ยวกับโครงการวิจัยแก่บุคคลที่เกี่ยวข้อง |  |  |  |  |  |  |  |  |  |  |  |  |  |
| ดำเนินการเก็บรวบรวมข้อมูล |  |  |  |  |  |  |  |  |  |  |  |  |  |
| วิเคราะห์ข้อมูล |  |  |  |  |  |  |  |  |  |  |  |  |  |
| เขียนรายงานการวิจัย |  |  |  |  |  |  |  |  |  |  |  |  |  |
| แก้ไขรายงานการวิจัย |  |  |  |  |  |  |  |  |  |  |  |  |  |
| เตรียมเสนอรายงานการวิจัย |  |  |  |  |  |  |  |  |  |  |  |  |  |

**งบประมาณ**

ค่าชาสมุนไพรบำรุงน้ำนมและชาใบเตย จำนวน 120 ชุด ได้รับการสนับสนุนจากเภสัชกรพินิต ชินสร้อย และคณะแพทย์แผนไทยจากรพ.วังน้ำเย็น

ค่าจ้างเหมาการเก็บข้อมูลอาสาสมัคร 120 คน คนละ 50 บาท คิดเป็นเงิน 6,000 บาท

ค่ายา Domperidone 1,000 เม็ดเป็นเงิน 2,000 บาท

ค่ายาหลอก Domperidone 1,000 เม็ด เป็นเงิน 2,000 บาท

ค่าอุปกรณ์สำนักงานและถ่ายเอกสาร 3,000 บาท

รวมค่าใช้จ่ายทั้งสิ้น 13,000 บาท

**เอกสารอ้างอิง**

1. Osadchy A, Moretti ME, Koren G. Effect of domperidone on insufficient lactation in puerperal women: a systematic review and meta-analysis of randomized controlled trials. ObstetGynecol Int. 2012; 2012: 642893.
2. Johannes CB, Varas-Lorenzo C, McQuay LJ, Midkiff KD, Fife D. Risk of seriousventricular arrhythmia and sudden cardiac death in a cohort of usersof domperidone: a nested case-control study. Pharmacoepidemiol Drug Saf2010;19:881-8.
3. Van Noord C, Dieleman JP, van Herpen G, Verhamme K, Sturkenboom MC.Domperidone and ventricular arrhythmia or sudden cardiac death: a populationbasedcase-control study in the Netherlands. Drug Saf2010; 33: 1003-14.
4. Zuppa AA, Sindico P, Orchi C, Carducci C, Cardiello V, Romagnoli C, et al. Safety and efficacy of galactogogues: substances that induce, maintain and increase breast milk production. J Pharm PharmaceutSci2010; 13:162-74.
5. พรทิพย์ เติมวิเศษ.การดูแลสุขภาพหญิงหลังคลอดด้วยการแพทย์แผนไทย. พิมพ์ครั้งที่ 2. ม.ป.ท. : กิจการโรงพิมพ์ องค์การสงเคราะห์ทหารผ่านศึก ; 2009: 103-104
6. Budzynska K, Gardner ZE, Dugoua JJ, Low Dog T, Gardiner P. Systematic review of breastfeeding and herbs. Breastfeed Med 2012; 7: 489-503.
7. จักรกฤษณ์ สุรการ, พินิต ชินสร้อย, วาสนา ชำนาญอักษร, เนตรนะภิส สุภะกะ, สาวิตรี งามวงศ์. ผลของชาสมุนไพรบำรุงน้ำนมต่อปริมาณน้ำนมและระดับโปรแลคตินในเลือดของมารดาหลังคลอด. พฤศจิกายน พ.ศ. 2554 (unpublished data)
8. [Wan EW](http://www.ncbi.nlm.nih.gov/pubmed/?term=Wan%20EW%5BAuthor%5D&cauthor=true&cauthor_uid=18507654), [Davey K](http://www.ncbi.nlm.nih.gov/pubmed/?term=Davey%20K%5BAuthor%5D&cauthor=true&cauthor_uid=18507654), [Page-Sharp M](http://www.ncbi.nlm.nih.gov/pubmed/?term=Page-Sharp%20M%5BAuthor%5D&cauthor=true&cauthor_uid=18507654), [Hartmann PE](http://www.ncbi.nlm.nih.gov/pubmed/?term=Hartmann%20PE%5BAuthor%5D&cauthor=true&cauthor_uid=18507654), [Simmer K](http://www.ncbi.nlm.nih.gov/pubmed/?term=Simmer%20K%5BAuthor%5D&cauthor=true&cauthor_uid=18507654), [Ilett KF](http://www.ncbi.nlm.nih.gov/pubmed/?term=Ilett%20KF%5BAuthor%5D&cauthor=true&cauthor_uid=18507654). Dose-effect study of domperidone as a galactagogue in preterm mothers with insufficient milk supply, and its transfer into milk.[Br J ClinPharmacol.](http://www.ncbi.nlm.nih.gov/pubmed/18507654) 2008;66:283-9.
9. Pierro J, Abulaimoun B, Roth P, Blau J. Factors associated with supplemental formula feeding of breastfeeding infants during postpartum hospital stay. Breastfeed Med 2016; 11: 196-202.
10. Surasak Jantarasaengaram, Praweena Sreewapa. Effect of domperidone on augmentation of lactation following cesarean delivery at full term:a randomized, double-blind, placebo-controlled trial.International Journal of Gynecology and obstestrics 2012; 116:240-243

**ภาคผนวก ก**

**หนังสือแสดงความยินยอมเข้าร่วมในการวิจัย**

หนังสือแสดงความยินยอมเข้าร่วมในการวิจัย

(Informed Consent Form)

วันที่……เดือน………………พ.ศ………..

ข้าพเจ้า…………………………………………………………………………………….. อายุ………………ปี

อาศัยอยู่บ้านเลขที่………….ถนน……………………แขวง/ตำบล………………..เขต/อำเภอ………………….

จังหวัด……………………….รหัสไปรษณีย์…………………โทรศัพท์……………………………………………

ขอแสดงเจตนายินยอมเข้าร่วมโครงการวิจัยเรื่อง “การศึกษาเปรียบเทียบประสิทธิภาพของ domperidone กับชาสมุนไพรในการกระตุ้นน้ำนมมารดาหลังผ่าตัดคลอดบุตร ”

โดยข้าพเจ้าได้รับทราบรายละเอียดเกี่ยวกับที่มาและจุดมุ่งหมายในการทำวิจัย รายละเอียดขั้นตอนที่จะได้รับการปฎิบัติประโยชน์ที่คาดว่าจะได้รับของการวิจัย และความเสี่ยงที่อาจจะเกิดขึ้นจากการเข้าร่วมการวิจัย โดยได้อ่านข้อความที่มีรายละเอียดอยู่ในเอกสารชี้แจงผู้เข้าร่วมการวิจัยเป็นที่เรียบร้อยแล้ว

หากข้าพเจ้ามีข้อข้องใจเกี่ยวกับขั้นตอนการวิจัยสามารถติดต่อกับ นพ.กุลชาติ แซ่จึง สังกัด รพ.สรรพสิทธิประสงค์ จ.อุบลราชธานี โทร.081-3924761

ข้าพเจ้าได้รับทราบสิทธิ์ที่ข้าพเจ้าจะได้รับทั้งด้านประโยชน์และโทษของการเข้าร่วมวิจัย และสามารถถอนตัวหรืองดเข้าร่วมการวิจัยได้ทุกเมื่อโดยไม่ต้องแจ้งให้ทราบล่วงหน้าหรือระบุเหตุผล โดยจะไม่มีผลกระทบต่อการบริการและการรักษาพยาบาลที่ข้าพเจ้าจะได้รับต่อไปในอนาคต และยินยอมให้ผู้วิจัยใช้ข้อมูลส่วนตัวของข้าพเจ้าที่ได้รับจากการวิจัย แต่จะไม่มีการเผยแพร่ต่อสาธารณะเป็นรายบุคคลโดยเด็ดขาด โดยจะนำเสนอเป็นข้อมูลโดยรวมจากการวิจัยเท่านั้น

ข้าพเจ้าได้เข้าใจข้อความในเอกสารชี้แจงผู้เข้าร่วมการวิจัย และหนังสือแสดงเจตนายินยอมนี้โดยตลอดแล้วจึงลงลายมือชื่อไว้

ลงชื่อ………………………………………………………ผู้เข้าร่วมวิจัย วันที่……………………………………

(……………………………………………………..)

ลงชื่อ………………………………………………………ผู้ให้ข้อมูล วันที่……………………………………

(……………………………………………………..)

ในกรณีที่ผู้เข้าร่วมวิจัยอ่านหนังสือไม่ออกผู้ที่อ่านข้อความทั้งหมดแทนผู้เข้าร่วมการวิจัยคือ……………………..

จึงได้ลงลายมือชื่อไว้เป็นพยาน

ลงชื่อ………………………………………………………พยาน วันที่……………………………………

(……………………………………………………..)

**ภาคผนวก ข**

**เอกสารชี้แจงผู้เข้าร่วมวิจัย**

**เอกสารชี้แจงผู้เข้าร่วมวิจัย**

**(Participant Information Sheet)**

ในเอกสารนี้อาจมีข้อความที่ท่านอ่านแล้วไม่เข้าใจโปรดสอบถามผู้วิจัยหรือผู้แทน ให้ช่วยอธิบายให้ท่านเข้าใจได้

**ชื่อโครงการ** : **ประสิทธิผลของชาสมุนไพรบำรุงน้ำนม (รพ.วังน้ำเย็น) ในการกระตุ้นการผลิตน้ำนมมารดาหลังคลอดบุตร (Efficacy of Wang Nam Yen Herbal Tea on Breast Milk Production : A Factorial Randomized Controlled Trial (Tea4Milk))**

**ผู้ดำเนินการวิจัย** : ชื่อ นายแพทย์กุลชาติ แซ่จึง ตำแหน่ง นายแพทย์ปฎิบัติการ กลุ่มงานสูตินรีเวชกรรม รพ.สรรพสิทธิประสงค์

**สถานที่วิจัย** : โรงพยาบาลสรรพสิทธิประสงค์ จ.อุบลราชธานี

**ระยะเวลาในการวิจัย** : 12 เดือน

**เหตุผลความเป็นมา**

ในประเทศไทยตั้งแต่อดีตมาหญิงหลังคลอดมีความสนใจเรื่องอาหารเพิ่มน้ำนม หรือหาซื้อยาแผนปัจจุบัน ยาสมุนไพรทั้งไทยและจีน มารับประทานเองเพื่อให้ปริมาณน้ำนมเพิ่มมากขึ้นให้เพียงพอต่อความต้องการของทารก การศึกษาในต่างประเทศรายงานว่ายา domperidone สามารถใช้เป็นสารกระตุ้นน้ำนมได้ domperidone เป็นสารกระตุ้นน้ำนมชนิดเดียวที่มี randomized controlled trial พบว่ามีประสิทธิภาพและมีความปลอดภัยในการใช้เพิ่มปริมาณน้ำนม1 แต่เมื่อเร็วๆนี้องค์การอาหารและยาของสหรัฐอเมริกา (USFDA) มีคำเตือนเกี่ยวกับการใช้ยาชนิดนี้ที่ให้ทางหลอดเลือดดำ อย่างไรก็ตามยังไม่มีรายงานผลเสียต่อมารดาหลังคลอดและลูกที่ได้รับยานี้โดยการรับประทาน การใช้ยาสมุนไพรไทยจึงเป็นอีกทางเลือกหนึ่ง

การใช้ยาสมุนไพรหลังคลอดมีวัตถุประสงค์หลายอย่าง เช่น เพื่อขับน้ำคาวปลา ขับเลือดเสีย ช่วยให้มดลูกเข้าอู่เร็ว บำรุงร่างกาย บำรุงน้ำนม ส่วนใหญ่จะมีฤทธิ์เผ็ดร้อน เนื่องจากในการแพทย์แผนไทยมีความเชื่อว่า ร่างกายของคนเราประกอบด้วยธาตุ 4 ได้แก่ ธาตุดิน ธาตุน้ำ ธาตุไฟ และธาตุลม โดยยึดหลักความสมดุลของธาตุทั้ง 4 เมื่อใดก็ตามที่เกิดความแปรปรวนจะมีความโน้มเอียงให้เกิดโรคหรืออาการที่ผิดปกติได้ ในภาวะหลังคลอดธาตุทั้ง 4 อยู่ในภาวะไม่สมดุล เนื่องจากการคลอดต้องใช้แรงเบ่ง ร่างกายสูญเสียน้ำ เสียเลือด อ่อนเพลีย ธาตุน้ำและธาตุลมในร่างกายเสียสมดุล ยังผลให้ธาตุไฟหย่อน การกินยารสร้อนจะช่วยให้ร่างกายอบอุ่น เป็นการบำรุงธาตุไฟ เมื่อธาตุไฟบริบูรณ์จะส่งผลให้ระบบต่างๆของร่างกายกลับคืนสู่สภาวะปกติได้เร็วขึ้น

การใช้สมุนไพรในการกระตุ้นน้ำนมเป็นที่นิยมทั่วโลก6 มีการใช้อย่างแพร่หลายตามค่านิยมและวัฒนธรรมท้องถิ่นมาเป็นเวลานานโดยไม่พบว่ามีอันตรายและมีประสิทธิภาพดี ทั้งที่ยังไม่ทราบกลไกการออกฤทธิ์แน่ชัด จนถึงปัจจุบันมีงานวิจัยเกี่ยวกับสมุนไพรไทยในการกระตุ้นน้ำนมน้อยมาก จากการศึกษาของ นพ.จักรกฤษณ์ สุรการ เรื่องชาสมุนไพรบำรุงน้ำนม ที่มีส่วนผสมของฝาง ชะเอม มะตูม ขิง และเถาวัลย์เปรียง ในมารดาหลังคลอด พบว่าได้ผลดีในกระตุ้นน้ำนมเมื่อเทียบกับยาหลอกและมีความปลอดภัยต่อมารดาและทารก ผู้ทำวิจัยจึงมีความสนใจที่จะทำการศึกษาต่อเนื่องจากงานวิจัยเดิมเพื่อเปรียบเทียบประสิทธิผลของชาสมุนไพรกับ Domperidone ซึ่งเป็น galactogue ที่นิยมใช้กัน เพื่อประโยชน์ในการดูแลมารดาหลังคลอดบุตรที่มีปัญหาน้ำนมไม่เพียงพอต่อไป โดยเลือกใช้ขนาดยา 30 mg/day เนื่องจากมีหลักฐานการใช้ยาในระดับสูงกว่านี้ไม่ได้เพิ่มปริมาณน้ำนม8 แต่อาจเพิ่มความเสี่ยงต่อการเกิดผลข้างเคียงได้ และเลือกกลุ่มประชากรที่จะศึกษาเฉพาะในมารดาหลังผ่าตัดคลอด อันเป็นหนึ่งใน ปัจจัยหลายประการที่ทำให้หญิงหลังคลอดมีปริมาณน้ำนมไม่เพียงพอต่อความต้องการของทารก

**วัตถุประสงค์ของการศึกษา**

**วัตถุประสงค์หลัก**

เพื่อศึกษาเปรียบเทียบผลของชาสมุนไพรบำรุงน้ำนมและ domperidone ต่อปริมาณน้ำนมของมารดาหลังผ่าตัดคลอดบุตร ณ 3 วันแรกหลังคลอด

**วัตถุประสงค์รอง**

1. เพื่อศึกษาเปรียบเทียบผลของชาสมุนไพรบำรุงน้ำนมและ domperidone ต่อระดับ prolactin ในกระแสเลือดของมารดาหลังผ่าตัดคลอดบุตร 3 วันแรกหลังคลอด

2. เพื่อศึกษาเปรียบเทียบผลข้างเคียงของชาสมุนไพรบำรุงน้ำนมและ domperidone ต่อมารดาหลังผ่าตัดคลอดบุตร เช่น ปากแห้ง ปวดศีรษะ ปวดท้องเป็นต้น

3. เพื่อศึกษาเปรียบเทียบผลของชาสมุนไพรบำรุงน้ำนมและ domperidone ต่อทารก เช่น น้ำหนักตัวที่เพิ่มขึ้นหรือลดลง การเกิดภาวะแทรกซ้อนเช่นตัวเหลือง เป็นต้น

4. เพื่อศึกษาเปรียบเทียบผลของชาสมุนไพรบำรุงน้ำนมและ domperidone ต่อมารดาที่ให้นมบุตร เช่น น้ำหนักตัว ภาวะแทรกซ้อน เช่น การตกเลือดหลังคลอด การติดเชื้อหลังคลอด เป็นต้น

5. เพื่อศึกษาความพึงพอใจของมารดาหลังผ่าตัดคลอด ที่ได้รับชาสมุนไพรบำรุงน้ำนมและ domperidone

**จำนวนอาสาสมัครที่คาดว่าจะเข้าร่วมโครงการ**

120 คน

**วิธีการรักษาหรือการปฏิบัติที่ใช้ในการวิจัย**

วิธีการรักษาหรือการปฏิบัติที่ใช้ในการวิจัยที่แตกต่างจากการปฏิบัติในงานปกติ

ในการวิจัยต้องการศึกษา ผลของยาต่อปริมาณน้ำนม รวมถึงภาวะแทรกซ้อนของมารดาและทารก โดยจะเริ่มปั้มนมด้วยเครื่องปั้มนม 12-72 ชั่วโมงหลังได้รับยา รวมทั้งบันทึกผลข้างเคียงระหว่างการให้ยา ได้แก่ ปากแห้ง ปวดหัว นอนไม่หลับ ปวดท้อง ท้องเสีย คลื่นไส้และปัสสาวะไม่ออก เป็นต้น พร้อมกับสอบถามความพึงพอใจและผลข้างเคียงเพิ่มเติม โดยจะไม่เกี่ยวข้องหรือมีผลต่อการวินิจฉัยหรือการรักษาที่ท่านได้รับ

**การเก็บตัวอย่าง**

เนื่องจากในงานวิจัยมีผลลัพธ์ที่ต้องการ คือ ปริมาณน้ำนม จึงขออนุญาตผู้ร่วมวิจัยในการปั้มนมด้วยเครื่องปั้มนม 12-72 ชั่วโมงหลังได้รับยา ซึ่งจะไม่มีผลเสียใดๆต่อทารกหรือมารดา พร้อมกับสอบถามความพึงพอใจและผลข้างเคียงเพิ่มเติม

สำหรับข้อมูลดังกล่าว จะมีเพียงแพทย์พยาบาลและทีมดูแลรักษาเท่านั้นที่ทราบข้อมูลและจะนำไปใช้เพื่อประโยชน์ในการติดตามรักษาและวิจัยเท่านั้น

**ผลเสียที่อาจได้รับในการเข้าร่วมโครงการวิจัยนี้มีอะไรบ้าง**

-ท่านอาจรู้สึกอึดอัดใจ รู้สึกไม่สะดวกสบาย เนื่องจากต้องกินยาเพิ่มทุกวัน เช้า กลางวัน เย็น และผู้ที่ได้รับชาสมุนไพรอาจมีปัญหาเรื่องการรับประทานเนื่องจากรสชาติ และลักษณะทางกายภาพของยาได้

-ท่านต้องเข้ารับการปั้มเต้านมด้วยเครื่องปั้มนม ทำให้รู้สึกอึดอัดที่ต้องมาทำการปั้มเต้านม อาจเจ็บเต้านมขณะทำการปั้มได้ แต่อย่างไรก็ตาม ในขณะปั้มเต้านม จะทำในห้องปิดมิดชิด และทำการตรวจวัดโดยเจ้าหน้าที่ผู้ชำนาญการและมีประสบการณ์ในการใช้เครื่องปั้มนม

**ประโยชน์ที่อาจได้รับ**

1.นำไปสู่การดูแลหญิงตั้งครรภ์ที่มีปัญหาเรื่องน้ำนมไม่เพียงพอ เพื่อเพิ่มปริมาณน้ำนมให้เพียงพอต่อความต้องการของทารก

2.เป็นแนวปฏิบัติสำหรับเจ้าหน้าที่สาธารณสุขในการนำสมุนไพรของไทยซึ่งหาได้ง่าย ผลิตเองได้ ราคาถูก มาใช้ในการส่งเสริมการเลี้ยงลูกด้วยนมแม่ให้ประสบผลสำเร็จ

3.เป็นแนวทางของผู้บริหารและหน่วยงานที่เกี่ยวข้องในการนำข้อมูลและความรู้ที่ได้จากการวิจัยนำมาปรับปรุง กำหนดนโยบายตลอดจนวางแผนการดำเนินงานส่งเสริมการเลี้ยงลูกด้วยนมแม่และพัฒนาการแพทย์แผนไทยให้ประสบผลสำเร็จ

**ข้อปฏิบัติของท่านขณะที่ร่วมในโครงการวิจัย**

ผู้วิจัยจะขอให้ท่านปฏิบัติดังนี้

ขอให้ท่านให้ข้อมูลทางการแพทย์ของท่านทั้งในอดีต และปัจจุบัน แก่ผู้วิจัยด้วยความสัตย์จริง

**ค่าใช้จ่ายสำหรับท่าน**

ท่านไม่ต้องเสียค่าใช้จ่ายใดๆ ในการรับบริการ หรือการตรวจทางห้องปฏิบัติการต่างๆ ตามที่ระบุไว้ในโครงการวิจัย

**การเข้าร่วมและการสิ้นสุดการเข้าร่วมโครงการวิจัย**

การเข้าร่วมในโครงการวิจัยครั้งนี้เป็นไปโดยความสมัครใจ ท่านอาจจะตัดสินใจไม่เข้าร่วมโครงการก็ได้ ท่านสามารถถอนตัวได้ตลอดเวลา ด้วยเหตุผลใดๆ ก็ตาม การขอถอนตัวออกจากโครงการวิจัยจะไม่มีผลต่อการดูแลรักษาโรคของท่านที่ได้รับอยู่ในปัจจุบันหรือในอนาคตแต่อย่างใด

**การปกป้องรักษาข้อมูลความลับ**

โครงการจะพยายามทำทุกวิถีทางเพื่อปกป้องความเป็นส่วนตัวของท่านและจะเก็บข้อมูลส่วนตัวของท่านเป็นความลับ การตีพิมพ์และเผยแพร่ผลการวิจัยจะไม่ระบุชื่อหรือข้อมูลส่วนตัวของท่าน

จากการลงนามยินยอมของท่าน ผู้วิจัย ตัวแทนของผู้สนับสนุนการวิจัย คณะกรรมการจริยธรรมการวิจัยในมนุษย์ของโรงพยาบาลสรรพสิทธิประสงค์ ผู้วิจัย และผู้ติดตามประเมินโครงการวิจัย สามารถเข้าไปตรวจสอบบันทึกข้อมูลทางการแพทย์ของท่านได้

หากท่านขอยกเลิกการให้คำยินยอมหลังจากที่ท่านได้เข้าร่วมโครงการวิจัยแล้ว ข้อมูลส่วนตัวของท่านจะไม่ถูกบันทึกเพิ่มเติม อย่างไรก็ตามข้อมูลอื่นๆ ของท่านอาจถูกนำมาใช้เพื่อประเมินผลการวิจัย

จากการลงนามยินยอมของท่าน ผู้วิจัยสามารถบอกรายละเอียดของท่านที่เกี่ยวกับการเข้าร่วมโครงการวิจัยนี้ให้แก่แพทย์ผู้รักษาท่านได้ เพื่อประโยชน์ของท่านเอง

**ภาคผนวก ค**

**แบบฟอร์มการเก็บข้อมูล**

**Case record form**

Case No.___________________________________Enrolled date____________________________

อายุ___________ปี อาชีพ_______________________________รายได้______________บาทต่อเดือน

สิทธิการรักษา__________________ ระดับการศึกษาสูงสุด__________________ศาสนา_______________

ลักษณะการเลี้ยงดูบุตร เลี้ยงบุตรคนเดียว เลี้ยงบุตรด้วยกันกับสามี มีญาติช่วยเลี้ยงดูบุตร

มีประสบการณ์ Breastfeeding ในท้องก่อน ไม่มี มี

G______P______A_______GA_________weeks

ANC at____________________จำนวน____________ครั้ง Hct จาก lab IIหรือ stat ________________

โรคประจำตัว_______________________________________ยาที่ใช้ประจำ______________________

ประวัติผ่าตัดอื่นๆ____________________________________แพ้ยา____________________________

Complication/Obstetric risks__________________________________________________________

Chief complaint_____________________________________________________________________

C/S due to _________________________________ วันที่____________เวลา________ EBL________ml

ทารกหนัก ___________ gm, Apgar ___________ เพศ____________________________ปัญหา____________________________________

NICU admission ไม่ มี เป็นเวลา____________วัน

รวมระยะเวลานอนรพ.ทารก____________________ รวมระยะเวลานอนรพ.มารดา______________________

I/O ใน 24 ชั่วโมงแรก_____________________ml

I/O จากการตวง ช่วง 24-48 ชั่วโมงหลังคลอด ________________ml

I/O จากการตวง ช่วง 48-72 ชั่วโมงหลังคลอด___________________ml

น้ำหนักตัวล่าสุดก่อนคลอด ____________หลังคลอด 72 ชั่วโมง____________ หลังคลอด 1 เดือน___________

เริ่มยาวันที่______________________เวลา_______________

ปริมาณน้ำนมที่ปั๊มได้ วันที่________เวลา__________ เต้าซ้าย______________เต้าขวา____________ml

น้ำหนักทารกก่อนกลับบ้าน__________________kg น้ำหนักมารดาก่อนกลับบ้าน_________________kg

Contraception ยังไม่ได้คุม ได้คุมโดย________________________

| หลังคลอด | วันที่ 0 | วันที่ 1 | วันที่ 2 | วันที่ 3 |
| --- | --- | --- | --- | --- |
| ปริมาณน้ำดื่มเฉลี่ย (แก้วต่อวัน) |  |  |  |  |
| จำนวนปัสสาวะเฉลี่ย  (ครั้งต่อวัน) |  |  |  |  |
| จำนวนครั้งที่ให้นมบุตรต่อวัน |  |  |  |  |
| ปริมาณน้ำนมเฉลี่ยต่อครั้งต่อเต้า (ml)  ข้างซ้าย/ข้างขวา |  |  |  |  |
| ปริมาณน้ำนมเพียงพอต่อความต้องการบุตรหรือไม่ | ___เพียงพอ  ___ไม่เพียงพอ | ___เพียงพอ  ___ไม่เพียงพอ | ___เพียงพอ  ___ไม่เพียงพอ | ___เพียงพอ  ___ไม่เพียงพอ |
| ต้องเสริมนมกระป๋องเฉลี่ย (ml ต่อวัน) |  |  |  |  |
| ต้องเสริมนมกระป๋องโดยประมาณกี่%ของปริมาณนมทั้งหมด |  |  |  |  |
| อาการข้างเคียง เช่น ปากแห้ง ปวดศีรษะ นอนไม่หลับ ปวดท้อง ท้องเสีย คลื่นไส้ ปัสสาวะไม่ออก |  |  |  |  |

**ส่วนนี้ประเมินเมื่อครบ 3 วัน ก่อนกลับบ้าน**

วัด V/S มารดา : BP___________PR_____________BT_____________RR______________

ระดับความสูงของยอดมดลูก _____________________________

สีของน้ำคาวปลา (lochia) สีแดงสด สีแดงจาง สีขาว

ภาวะแทรกซ้อนของมารดา :

การตกเลือดหลังคลอด ไม่มี มี immediate PPH delayed PPH

การติดเชื้อหลังคลอด
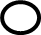
 ไม่มี
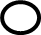
 มี ระบบ_____________________

ภาวะแทรกซ้อนอื่นๆ(ระบุ) ________________________________________________

ภาวะแทรกซ้อนของทารก :

ตัวเหลือง ไม่มี มี

หายใจผิดปกติ ไม่มี มี

ภาวะแทรกซ้อนอื่นๆ (ระบุ)__________________________________________________

การใช้ยาอื่นเสริม : ไม่มี มี เช่น____________________________________

การกินอาหารที่ทำให้ปริมาณน้ำนมเพิ่มขึ้น ไม่มี มี เช่น_____________________________________

| ความเห็นด้วย | น้อยที่สุด | น้อย | ปานกลาง | มาก | มากที่สุด |
| --- | --- | --- | --- | --- | --- |
| ความพึงพอใจต่อการให้นมบุตร |  |  |  |  |  |
| ความพึงพอใจในการใช้ยากระตุ้นน้ำนม |  |  |  |  |  |
| ความกังวลเรื่องปัญหาการให้นมบุตร |  |  |  |  |  |
| ความกังวลต่อผลข้างเคียงของยา |  |  |  |  |  |
| การตัดสินใจใช้ยาต่อไป(อัตราการคงใช้) |  |  |  |  |  |
| จะแนะนำเพื่อนที่รู้จักให้ใช้ยาเพื่อช่วยกระตุ้นน้ำนม |  |  |  |  |  |
| จะแนะนำเพื่อนที่รู้จักให้อาหารเพื่อเพิ่มปริมาณน้ำนม |  |  |  |  |  |
| จะแนะนำเพื่อนที่รู้จักเกี่ยวกับวิธีการให้นมบุตรที่ถูกต้อง |  |  |  |  |  |
| จะใช้ยากระตุ้นน้ำนมอีกในการตั้งครรภ์ครั้งหน้า |  |  |  |  |  |
