# Supplementary material for: Efficacy of Wang Nam Yen herbal tea on human milk production: A randomized controlled trial
Source: PLoS One. 2022 Jan 31;17(1):e0247637. doi: 10.1371/journal.pone.0247637 (PMC8803155; doi:10.1371/journal.pone.0247637)
Supplement: Attachment — Submitted filename: Response to Reviewer Tea4Milk 8.2 (PLoS One) KP clean.docx [file pone.0247637.s005.docx]

Dear Editor,

We thank you and the reviewers for the comments and suggestions. We have tried our best to address all of the points raised by you and the reviewers during the past 12 days offered. Please find our point-by-point responses below:

Editor: Thank you for submitting your manuscript to PLOS ONE. After careful consideration, we feel that it has merit but does not fully meet PLOS ONE’s publication criteria as it currently stands. Therefore, we invite you to submit a revised version of the manuscript that addresses the points raised during the review process.

Response: Thank you for your time and consideration. The manuscript was revised to meet PLOS ONE’s publication criteria.

Editor: There are a number of issues pointed out by the reviewers. Reviewer 2 has put in a lot of effort and the comments are very useful and if addressed could add a lot of value to the manuscript. Please address all of these either by editing the submission or explaining why you didn't. One of the most pertinent issues is about the onset of action following ingestion of the two galactagogues in the study. Is anything know about how long it would take for the tea to act and at which stage of lactation it has its main effect? More information would be helpful for the reader to understand whether the time from first ingestion to the time of measurement of milk volume is sufficient and also whether a similar effect might be seen if used later in lactation.

Response: Thank you for your comment. We greatly appreciate Reviewer 2 for the useful pieces of advice. The information about the onset of action following ingesting of the two galactagogues was added in the Introduction section.

Editor: The issue of inclusion of preterm infants is an important one. It would be helpful to give more information about the preterm infants in the study such as the proportion of preterm infants, very preterm infants and full term infants. This would help readers to judge how much impact the inclusion of preterm infants had on the results. Even with the galactagogue the volume of milk expressed is likely to be lower than in a term infant.

Response: We are sorry for the confusing inclusion criteria and discussion. Initially, we intended to include both preterm and term pregnant women, but they were separated from their child before participation which was one of the exclusion criteria. Hence, all included participants were term pregnant (Gestational age 37–42 weeks). To minimize confusion, the inclusion criteria were amended from the first registered protocol. The inclusion criteria in the Methods section were revised and the reason for inclusion criteria amendment was provided in the Discussion section.

Editor: The reviewers were also confused by your use of the terms Day 4 and 3-days. I assume Day 4 means that infant was 3-days old and not yet 4-days old. i.e 72-48 hours of age? I think it would be useful to just use hours of life or a range of hours such as 72-96 hours.

Response: Thank you for pointing out and sorry for the confusion. The terms were revised for clarification throughout the manuscript.

Editor: It would be useful to the reader if you gave more information in the section on statistical analysis about the use of the Bonferroni correction. With the correction in place what p-value was assumed significant?

Response: An additional information on Bonferroni correction was added in the Methods section. P-value was assumed significant at p < 0.017.

Editor: 1. Please ensure that your manuscript meets PLOS ONE's style requirements, including those for file naming.

Response: The manuscript was revised to meet PLOS ONE’s style requirements.

Editor: 2. In order to meet our requirements for data accessibility, please provide the following information about the Wang Nam Yen herbal tea prepared by the hospital and the control herbal tea: a) the sources of all ingredients, including the full name of the company or local market; b) the lot numbers of the ingredients; c) the amount of each ingredient contained in one dose of tea

Response: All ingredients for the Wang Nam Yen herbal tea and the control herbal tea were from Charoensuk
Pharma Supply Co.,Ltd. (<https://www.cpspharma.co.th/>). Unfortunately, no lot number was provided by the company. Each Wang Nam Yen herbal tea bag contained 500 mg sappan (Caesalpinia sappan Linn.), 500 mg licorice (Glycyrrhiza glabra Linn.), 500 mg bale fruit (Aegle marmelos L. Corr), 500 mg ginger (Zingiber officinale Roscoe), and 500 mg jewel vine (Derris scandens (Roxb.) Benth). The herbal tea placebo was also prepared as a teabag for each meal, and the placebo tea bag was identical to Wang Nam Yen teabag. The placebo tea bag was filled with 2,500 mg pandan (Pandanus amaryllifoilus) leaves. The information was provided in the Methods section.

Editor: 3. Please provide additional details regarding participant consent. In the ethics statement in the Methods and online submission information, please ensure that you have specified whether you obtained written informed consent from parents or guardians of 15-17 year old participants.

Response: Additional details on participant consent were added in the Methods and online submission information.

Editor: 4. We note that you have indicated that data from this study are available upon request. PLOS only allows data to be available upon request if there are legal or ethical restrictions on sharing data publicly. For information on unacceptable data access restrictions, please see http://journals.plos.org/plosone/s/data-availability#loc-unacceptable-data-access-restrictions.

Response: There are no restrictions. De-identified data set was provided as a Supporting Information file.

Reviewer #1: This paper reports on a randomised trial of two approaches to breast milk production in women undergoing Caesarean delivery. There are a number of issues with this paper. The design is a three arm trial -the authors need to explain why a 2x2 design was not looked at as this would help see whether the effects of the interventions were additive.

Response: Thank you for pointing this out. The rationale for factorial design was that domperidone—the current standard medication for promoting lactation—is in form of an oral tablet while Wang Nam Yen herbal tea is in form of a teabag needed for infusion of water before use. Thus, the placebo oral tablet and placebo tea were necessary for this study. The 2x2 design was in our initial research proposal but one arm (herbal tea + domperidone) had to be discarded in response to the comment from the ethics committee. Hence, the potential additive/synergistic effect could not be inferred from our data.

Reviewer #1: The sample size is not given in sufficient detail to allow someone to reproduce it - especially as the primary comparison would be a 3-way one and the actual effect sizes and sd are not given in the paper.

Response: Thank you for your comment. The sample size calculation subheading was revised in the Method section to provide more reproducibility of the study.

Reviewer #1: The allocation method was via sealed envelopes presumably containing the allocation - the investigator is therefore not blind. A block size of three (presumably one per arm per block) gives the opportunity of significant treatment foreknowledge and as such the integrity of the randomisation process is not guaranteed. What tests were performed to ensure envelopes were opened in order and were those consenting women kept in ignorance of all previous treatment allocations? What were the reasons for not entering and did they differ by next allocation?

Response: Thank you for pointing this out. The randomization and blinding subheadings were revised in the Methods section to clarify the process. The allocation method was deliberately ensured that the investigator was blinded. That is, after generating randomization codes, the research team responsible for blinding created an opaque envelope to seal the randomization codes. The front end of an opaque sealed envelope presented the study name, principal investigator name, site of study, randomization ID, study ID, and date which envelope will be opened. There was a document inside the sealed envelope with randomization ID, assigned group, study ID, date and time which envelope will be opened, and signature of envelope opener who are not clinician nor data collector. While a single block size (of three) might cause potential treatment foreknowledge and lead to selection bias, in theory, this trial random allocation was done by a separate research team independent from enrollment personnel, clinician, or data collector to prevent the selection bias.

Reviewer #1: 72 hours is 3 days post delivery - why are we variously told about 4 day outcomes?

Response: We are sorry for the confusion. The terms were revised for clarification throughout the manuscript.

Reviewer #1: It is statistically incorrect to test at baseline (Table I).

Response: Thank you for your comment. The p-values presented in Table 1 were to demonstrate the comparable baseline characteristics of participants across the three groups after the randomization. We believe this statistical information is useful but could be removed as appropriate.

Reviewer #1: What methods are used when? ANOVA requires iid Normal residuals, Kruskal-Wallis would be used when this isn't the case. Why are they being performed simultaneously? Please explain the tests used at each point and give contrasts and CI wherever possible.

Response: Thank you for pointing out a concerning issue. ANOVA and Kruskal-Wallis were not performed simultaneously. ANOVA was used when the continuous variable was normally distributed while Kruskal-Wallis was used when the continuous variable was not normally distributed. The statistical analysis subheading in the Methods section was revised for clarification. Footnote on which statistical test was performed for each variable was added in Table 1.

Reviewer #1: Drug AEs are not assessed similarly between groups and need to be removed or assessed blind to allocation.

Response: The allocation concealment was deliberately performed to ensure double-blinded as stated above. Drug AEs were assessed blindly and similar among groups. The Methods section was revised to provide more detail and clarification on randomization and blinding, and outcome measurement.

Reviewer #2: This study is of good quality with appropriate controls and the authors have followed the guidelines recommended by CONSORT for RCT-studies. The authors have explained that all data relevant to the study have been included in the article and any de-identified data could be shared upon request. The text is quite well written. However, most of the text is written in past tense, and in certain areas, this can be misleading. An example is the first sentence in the introduction section. Perhaps the authors can re-check the whole manuscript to ensure that the tense used is appropriate.

Response: Thank you for the compliments. Appropriate use of tense was revised throughout the manuscript.

Reviewer #2: Introduction: i. To justify the measurement of the outcome at 72 hours, I suggest that the authors provide data for both Wang Nam Yen and domperidone (with appropriate reference to the literature) on how long after ingestion will a galactogenic effect be expected.

Response: Thank you for your comment. Additional details on the expected onset of lactogenic effect for both Wang Nam Yen and domperidone were added in the Introduction section.

Reviewer #2: Introduction: ii. A reference is needed for the last few sentences in the 2nd paragraph, and the first sentence of the 3rd paragraph of this section.

Response: Thank you for your comment. The references were added as suggested accordingly.

Reviewer #2: Introduction: iii. Do the authors have data on which component of the Wang Nam Yen is the most active galactagogue? Some discussion on this would be interesting.

Response: Thank you for your comment. Additional information on the most active galactagogue was added in the Introduction section.

Reviewer #2: Study design: i. It is not very clear when the participants were recruited. Was it before or after delivery?

Response: The participants were recruited after delivery by cesarean section. Additional detail was added in the Method section for clarification.

Reviewer #2: Study design: ii. Newborn-mother separation is one of the exclusion criteria. Please clarify why were the 6 mothers whose babies were admitted to NICU (thus meeting the exclusion criteria) included in the study.

Response: Study design: 6 babies were admitted to NICU after participation but did not separate from their mothers whereas the exclusion criteria excluded newborn-mother separation before participation. The Method section was revised to clarify the eligibility criteria.

Reviewer #2: Participants: i. Premature infants were included. Their sucking reflex and chances for direct breastfeeding could be impaired. The main stimulant for more breast milk production is direct breastfeeding and expression of breast milk which may not go well with premature infants. How did the authors manage this?

Response: We are sorry for the confusing inclusion criteria and discussion. Initially, we intended to include both preterm and term pregnant women, but they were separated from their child before participation which was one of the exclusion criteria. Hence, all included participants were term pregnant (Gestational age 37-42 weeks). To minimize confusion, the inclusion criteria were amended from the first registered protocol. The inclusion criteria in the Methods section were revised and the reason for inclusion criteria amendment was provided in the Discussion section.

Reviewer #2: ii. It would be important for readers to know the rationale behind why only mothers who had undergone caesarean section was included in this study.

Response: Women that have undergone cesarean deliveries were selected as the population in this study due to cesarean delivery was a risk factor of inadequate breast milk production. Additional detail was provided in the Introduction section.

Reviewer #2: Randomization and blinding: i. The authors have taken trouble to ensure each group received similar looking herbal tea bags and tablets. However, it would be important to mention if the intervention tea and the placebo tea will both have the same appearance, taste, and smell. The same goes for the tablets (domperidone vs placebo).

Response: Thank you for pointing it out. Additional detail on placebo tea and tablet was provided in the Methods section. While we intended to produce an ideal placebo tea, the taste and aroma of placebo tea were still different from the Wang Nam Yen herbal tea. However, no participants had a chance to take both active and placebo tea simultaneously. An additional discussion on this limitation was provided in the Discussion section.

Reviewer #2: ii. It would be important to mention what was actually used to fill in the placebo tea bags and what was the ingredient used in the placebo tablet, so that readers can be certain that these placebos do not have any galactogenic effect.

Response: The information of ingredients used in placebo tea bags and tablets were added in the Method section accordingly.

Reviewer #2: iii. Details of how the random sequence was generated and how the allocation concealment was done (which was nicely written in the protocol) should be added into the manuscript.

Response: The details of randomization and allocation concealment were added to the Methods section accordingly.

Reviewer #2: iv. Other important details available from the protocol such as the availability of similar breastfeeding support to all the groups should also be included into the manuscript.

Response: The Methods section was revised as suggested accordingly.

Reviewer #2: Intervention: i. The sentence "Domperidone was administered at 10 mg per dose, as 30 mg of domperidone per day" is rather confusing. Could this be written in as “Domperidone was given 10mg 3 times per day (total dose 30mg daily)."?

Response: The sentence was revised as suggested accordingly.

Reviewer #2: Outcomes measures: i. Clarification is needed on what was the unit of analysis used for the milk volume measurements. Was the recorded milk volume a single measurement of 'the total amount' obtained from both breasts after a single expression (mls/extraction) OR was it an accumulation of milk volume measured several times a day (mls/day)?

Response: Thank you for your comment. The unit of analysis was the total amount of milk volume in a single expression (mL/extraction). The Methods section was revised accordingly.

Reviewer #2: Outcomes measures: ii. I note that the milk expression is done 2 hours after the baby has last fed. Can the authors clarify how milk collection was done for babies that demand hourly feeding?

Response: The milk collection was done uniformly for all participants. While some babies might demand hourly feeding, all mothers were notified at 2 hours before each milk collection section which allowed the last breastfeeding prior to the milk collection. The Methods section was revised accordingly.

Reviewer #2: Outcomes measures: iii. Did the mother express her milk herself or did a trained staff help her to do it? This is to see how uniform the milk expression was done.

Response: A trained nurse helped all participants to express their milk. Additional detail was added to the Methods section accordingly.

Reviewer #2: Outcomes measures: iv. It would be important to describe how the babies were mainly fed in each group. Were the mothers mainly directly breastfeeding or were many of them expressing milk for feeding? It would be useful to know this proportion because frequent effective direct breastfeeding is a powerful stimulator for breast milk production and this could affect the end result.

Response: Thank you for your comment. Unfortunately, there was no recorded data on the proportion of mothers directly breastfeeding in each group. This was one of the limitation of the study and the Discussion section was revised accordingly.

Reviewer #2: Outcomes measures: v. Since supplementing feeding with formula can affect the amount of breastmilk produced, it would be important to mention how many babies were supplemented with milk in each group.

Response: Thank you for your comment. Information on formula milk supplement measure was added in the Methods and Results section.

Reviewer #2: Outcomes measures: vi. Formula milk supplementation might also affect the baby’s intake and cause less breast emptying, thus a false sense of ‘more breastmilk’. The authors could elaborate on how this is managed.

Response: The need for formula milk supplementation was recorded daily and categorized into 4 groups: no formula milk supplement, required 1-day supplement, required 2 days supplement, and required 3 days supplement. Association between intervention groups and formula milk supplementation was evaluated by Chi-square test. Additional details were added in the Result and the Discussion sections.

Reviewer #2: Outcomes measures: vii. Table 1 has lots of details such as method of payment, number of antenatal visits and presence of labour symptoms. Perhaps the authors could explain why these information were included. If they do not contribute to breast milk production i.e. potential confounders, perhaps they can omit these details so that the table is less cluttered.

Response: Most variables could be potential confounders of breast milk production while some variables were potential confounders for a secondary outcome such as the method of payment, presence of labor symptoms, low socioeconomics status could be potential confounders of postpartum endometriosis. Variable without evidence for potential confounders were omitted from Table 1 as advised.

Reviewer #2: Outcomes measures: viii. There is a statement "There were no significant differences between the groups in …….72 hours postpartum period or the amount of formula milk required (Table 1)." The amount of formula milk required was not presented in Table 1. I hope the authors will include this in the table.

Response: We apologize for the missing information in Table 1. The need for formula milk supplementation was recorded daily and categorized into 4 groups: no formula milk supplement, required 1-day supplement, required 2 days supplement, and required 3 days supplement. The missing information was added in Table 1 accordingly.

Reviewer #2: Results: i. Mothers with premature babies may have different milk volume production because of physiological reasons and difference in infant sucking abilities. Therefore, the number of mothers with premature babies in each group should be mentioned.

Response: As mentioned above, all included participants were term pregnant; no premature infants were included.

Reviewer #2: Results: ii. Being able to directly breastfeed a baby can make a difference in milk supply. It would be useful for the authors to provide data (if available) on the proportions of mothers directly breastfeeding in each group.

Response: Thank you for your comment. Unfortunately, there was no recorded data on the proportion of mothers directly breastfeeding in each group. This was one of the limitation of the study and the Discussion section was revised accordingly.

Reviewer #2: Results: iii. Do the authors have data on whether the participants have lactation insufficiency, and if so, how many were there in each group? It would be useful for this to be mentioned because lactation insufficient women may produce milk differently from lactation sufficient women. The author’s definition of what they consider lactation insufficient will also be needed if this data is included.

Response: Thank you for your comment. We did not evaluate lactation insufficiency in this study. I agree that lactation insufficiency might affect breast milk production. This was a limitation of the study and additional discussion was added in the Discussion section.

Reviewer #2: Results: iv. It would be better and clearer if the actual volume and SD (baseline and at 72 hours) were reported. If possible, display in a table. Showing odds ratio of linear regression (95% confidence interval) could be more meaningful than just the p value. Readers will be interested to know the values obtained to support the basis that taking Wang Nam Yen or Domperidone would make a meaningful difference.

Response: Thank you for your comment. The actual volume and SD of breast milk were added in Table 2.

Reviewer #2: Results: v. In addition, the results described narratively in the text and the results displayed in the graph did not seem to match and were confusing. The text stated that the milk volume in Group T was significantly higher than that in Group C, but in the graph, both Group T and Group D were plotted very closed to each other at 72 hours. On the other hand, the text stated that milk volume for Group D and C were not statistically different, but in the graph, they were obviously plotted very far from each other. Further clarification is needed.

Response: While the statistical test compared mean and SD, the graph plot presented mean with a 95% confidence interval. The distance between the groups in the graph presented a close distance between Group T and Group D while a far distance of Group T or Group D from Group C. The graph plot reflected statistically significant unadjusted results between Group T and Group C, Group D, and Group C. However, when Bonferroni correction was applied, the adjusted result was more valid and might differ from the graph plot.

Reviewer #2: Results: vi. The unit of analysis for Figure 2 is needed in the title of the figure. Was it a mean volume of milk per day at 72 hours of life or volume of milk per expression at 72 hours of life, or was it a mean increment of milk volume since baseline?

Response: Thank you for your comment. The unit of analysis (volume of milk per expression) was added as suggested accordingly.

Reviewer #2: Results: vii. The authors did mention that milk volume in the domperidone group showed no significant difference from the placebo group after applying Bonferroni correction. Please clarify what was meant by the phrase 'too conservative'. What confounders was used?

Response: An additional detail on Bonferroni correction was added in the Methods and Discussion sections. The disadvantage of Bonferroni's correction was small-than-required adjusted α, thus often caused false-negative result. ‘Too conservative’ is equivalent to ‘weak statistical power’. Potential confounders in this study were maternal age, socioeconomic status, method of payment, gestational age, parity status, adequate antenatal care, presence of labor symptom, previous cesarean section, the experience of breastfeeding, neonatal birth weight, postpartum hemorrhage, fluid balance, and formula milk required.

Reviewer #2: Results: viii. About the adverse effects and Table 2: Why was postpartum endometritis highlighted separately in Table 2? If this could be related to the intervention, perhaps it should be discussed in the discussion section as well?

Response: We highlighted postpartum endometritis because this complication was common in cesarean delivery. Information that Wang Nam Yen herbal tea will not significantly increase postpartum endometritis compared to domperidone and placebo could provide more clear safety profile of Wang Nam Yen herbal tea. An additional discussion was added in the Discussion section.

Reviewer #2: Results: I am confused with the actual number of adverse events. The text mentioned that 3 mothers suffered adverse events and only mentioned 3 types of adverse events, namely postpartum endometriosis, dry mouth and diarrhoea. However, Table 2 reported 6 types of adverse events (headache, dry mouth, diarrhea, muscle cramps, itching, or allergic reactions) and that there was a total of 6 mothers experiencing them. Could the authors clarify?

Response: We apologize for the confusing information. There were 3 mothers who suffered adverse events: one with postpartum endometriosis in the control group (we displayed this event in Maternal complication), one with dry mouth in the domperidone group, one with diarrhea in Wang Nam Yen herbal tea group. Table 2 legend “Drug adverse event includes headache, dry mouth, diarrhea, muscle cramps, itching, or allergic reactions.” was intended to mention which symptoms could be considered as drug adverse event, not the actual drug adverse events occurred.
Thus, Table 2 legend was revised to present actual drug adverse events only.

Reviewer #2: Results: The authors took the trouble to separate out adverse effects occurring at 24h, 48h and 72h in Table 2. Is there a reason to do so? If there is, perhaps details should then be provided as to which adverse effect occurred at which time, and discuss this in the discussion section as well?

Response: We recorded adverse events daily for two reasons. First, there was a lack of evidence on when the Wang Nam Yen herbal tea might cause adverse events. Second, we intended to assess how long the adverse events persisted and subsided. Additional details were added to the Results and the Discussion sections.

Reviewer #2: Results: ix. About the 6 infants that required NICU admission: What was the reason behind the 6 infants requiring ICU admission? How were these mother-infant pairs handled when separation did happen after randomization? How many were there in each group? Clear explanation and details would allow readers to know if the admissions were related to intervention and if intention to treat analysis was performed.

Response: The six infants were admitted to NICU due to respiratory distress syndrome or transient tachypnea of the newborn (TTNB) which the authors assumed no association with intervention. When mother-infant separation occurred after randomization, the mothers continued in the trial similar to other participants except direct breastfeeding was not possible while the infants received standard of care. There was 1 infant admitted to NICU in Wang Nam Yen herbal tea group, 2 in the domperidone group, and 3 in the placebo group. Additional details were added in the Methods, Results, and Discussion sections.

Reviewer #2: Results: x. The manuscript reported some participants experiencing adverse effects which subsided after discontinuing the intervention. Further details are needed to see how were they related to the intervention or could affect the analysis. How many other participants in each group did not actually complete the intervention as prescribed, ie did not take it 3 times a day for 3 days? At which time point did these participants in each group stopped taking the intervention? Was the milk expression continued at each time point for analysis? If yes, was the participants analysed based on their randomized group? Perhaps these details could be included in the flow diagram or at least in the text of the results.

Response: We apologize for the confusing information. The phrase “which subsided after discontinuing the intervention” means that the adverse symptoms persisted for 72 hours postpartum and then subsided after the trial end (no intervention received). Thus, all participants were complete the intervention as prescribed and analyzed based on their randomization group. The discussion on adverse events was revised in the Discussion section.

Reviewer #2: Discussion: i. The manuscript stated "Although some participants declined to enroll in the trial following randomization, …..". This line needs clarification because it contradicts the flow diagram that showed that participants who declined to enroll were not randomised. If these were participants who had originally consented to take part in the study, but pulled out after randomization, they should be reflected in the study flow diagram as participant drop-outs, hence the final number of participants who were analysed would be less than 40 in each group. The numbers in Table 2 and 3 will also need adjustments. The reasons for dropping out (if any) should be described.

Response: We apologize for the confusing information. While there were some participants who decline to enroll in the trial before randomization, there were no participants who declined after randomization. Relevant detail in the Discussion section was revised accordingly.

Reviewer #2: Discussion: ii. Data on confounding factors to milk production should be stated. This includes: when breastfeeding was first initiated (the protocol says that mothers were encouraged to breastfeed within 24 hours, but having her first breastfeed at birth or first breastfeed at 18 hours of life is a big confounder), babies were supplemented with formula in each group, number of infants with sucking problems, number of mothers with lactation insufficiency. If they were not available, they could be discussed as limitations of the study.

Response: We apologize for the confusing information. The first breastfeeding was first initiated after the first dose of intervention which occurred 12-18 hours following delivery. Encouragement to breastfeed their infant within 24 hours of delivery was Sanpasitthiprasong hospital's standard of care policy. The Methods section was revised accordingly. Need for formula milk supplementation was recorded daily and categorized into 4 groups: no formula milk supplement, required 1-day supplement, required 2 days supplement, and required 3 days supplement. Association between intervention groups and formula milk supplementation was evaluated by Chi-square test. Additional details were added in the Result and the Discussion sections. We did not evaluate sucking problems in this study. We agree that sucking problem might be a potential confounder of breast milk production. This was a limitation of the study and additional discussion was added in the Discussion section. Likewise, we did not evaluate lactation insufficiency in this study. I agree that lactation insufficiency might affect breast milk production. This was a limitation of the study and additional discussion was added in the Discussion section.

Reviewer #3: This is an interesting study that seeks to compare the effectiveness of Wang Nam Yen herbal tea vs. domperidone compared to placebo in achieving improved mother's milk production at 72 hrs following delivery. There were 120 participants in the trial who ranged in delivery gestational age between 28-40 weeks, with a mean GA of ~38 wks and a bw of ~3 kg. The treatment was prescribed for 72 hrs with milk volume measurements via pumped milk expression at each 24 hr interval. There were statistically significant differences between Wang Nam Yen herbal tea and placebo, but not between domperidone and placebo or between Wang Nam Yen herbal tea and domperidone. These are important findings that will serve as the basis for future studies. The following are points that should be addressed to strengthen the clarity and strength of the findings:

Response: Thank you for your compliments. The manuscript was revised as suggested accordingly.

Reviewer #3: (1) In the Methods, the placebo tea needs to be delineated and described. How were the participants and investigators blinded given that herbal teas with certain ingredients have particular aromas?

Response: Thank you for pointing it out. Additional detail on placebo tea and tablet was provided in the Methods section. While we intended to produce an ideal placebo tea, the taste and aroma of placebo tea were still different from the Wang Nam Yen herbal tea. However, no participants had a chance to take both active and placebo tea simultaneously. An additional discussion on this limitation was provided in the Discussion section.

Reviewer #3: (2) In the Methods, please describe the block size of randomization.

Response: Thank you for your comment. The block size of randomization and additional detail were added in the Methods section.

Reviewer #3: (3) Methods: in the sample size estimate, you mention milk volume at 4 days but this analysis was at 72 hrs. Perhaps you are using data for power that described milk volume differences at 4 days for your sample size calculation. Please rewrite this section.

Response: Thank you for pointing out a concerning issue. The sample size calculation subheading was revised in the Method section to provide more detail and reproducibility of the study.

Reviewer #3: (4) Methods: it is concerning that women who delivered preterm were analyzed with women who deliver term. I think this is a potential weakness of the study design. Further, in the analyses of neonatal side-effects, the study is not powered to look at preterm neonatal outcomes by treatment. The tea and domperidone may not differ in their safety profile with preterm mothers, but you would need far more mothers enrolled in such a study. I do not think you can extrapolate safety to those born <35wks so easily. You might suggest that this be refined in future studies.

Response: We are sorry for the confusing inclusion criteria and discussion. Initially, we intended to include both preterm and term pregnant women, but they were separated from their child before participation which was one of the exclusion criteria. Hence, all included participants were term pregnant (Gestational age 37-42 weeks). To minimize confusion, the inclusion criteria were amended from the first registered protocol. The inclusion criteria in the Methods section were revised and the reason for inclusion criteria amendment was provided in the Discussion section.

Reviewer #3: (5) The Discussion needs to be checked for accuracy for scientific English to make it more readable. There are grammatical errors in that section that are not seen in prior sections.

Response: Thank you for your comment. The Discussion section was revised was more readable scientific English.

We hope that our responses are satisfactory. Should there be anything that might improve our work, please kindly inform us. Thank you very much for your kind consideration.

Best Regards,

Assoc. Prof. Dr. Krit Pongpirul, MD, MPH, PhD.

On behalf of the authors
