# Supplementary material for: Efficacy of Wang Nam Yen herbal tea on human milk production: A randomized controlled trial
Source: PLoS One. 2022 Jan 31;17(1):e0247637. doi: 10.1371/journal.pone.0247637 (PMC8803155; doi:10.1371/journal.pone.0247637)
Supplement: Attachment — Submitted filename: Response to Reviewer Tea4Milk 2 KP clean.docx [file pone.0247637.s006.docx]

Dear Editor,

We thank you and the reviewers for the comments and suggestions. We have tried our best to address and clarify all of the 12 concerning points raised by you and the reviewers. Please find our point-by-point responses below:

Editor: I have been through the reviewer responses to your feedback and although they are attached I am highlighting below those areas that need to be addressed. Therefore please respond to these 12 points. If you would like to respond to other points made by the reviewers please do.

Response: Thank you for your time and consideration. We sincerely appreciate you and Reviewer 2 for the invaluable advice. The manuscript was revised to address all concerning issues and to meet PLOS ONE’s publication criteria.

Editor: 1. Introduction. Please go through the introduction carefully as some parts are still in the past tense while some parts of the methods are in future tense.

Response: Sorry for improper English grammar use. The introduction and methods sections were thoroughly revised to provide proper English grammar use.

Editor: 2. Sample size calculation. Your response does not really address the concern of the statistical reviewer. It appears that your sample size calculation is based on having 2 parallel groups whereas you have 3. Therefore is the sample size calculation floored? If this is true then it needs to be discussed as a serious limitation in the study

Response: There was a limitation in sample size calculation due to no published peer-reviewed evidence on the effect of this herbal tea. Thus, we calculated the sample size using data based on two parallel groups (domperidone and placebo). This might cause a potential problem due to underpower of the study if the true effect of herbal tea was significantly lower than that of domperidone. On the other hand, if the true effect of herbal tea was significantly higher than that of domperidone, there would be only overestimation of sample size. The Discussion section was revised to address this limitation.

Editor: 3. Randomisation Process. The concern is not about who prepared the envelopes, and here you have used an appropriate method, but rather whether the person recruiting patients and deciding whether they met the inclusion criteria know which group the previous patient recruited was allocated to? If they did then they may foreknowledge of what the next allocation will be and this could affect their decision whether to recruit a particular patient. I suggest you describe the recruitment process from this angle? Was recruitment always done by the same clinician? Would they know the previous allocation? Do you need to acknowledge a limitation here?

Response: Thank you for pointing out this important concern and sorry for the unclear message. The allocation seal envelop opener was the only person who knows which group the patient belongs due to the opener was not involve with the recruitment process, was not the clinician, and was not the data collector as described in the manuscript. This process was done to minimize the potential selection bias from a single block size. The Randomization and blinding section was revised as suggested whereas the limitation of single block size randomization and the method to minimize the potential selection bias was provided in the Discussion section.

Editor: 4. Participants. Inclusion of preterm infants. It is still not clear whether or not you intended to include preterm infants. In the methods it appears that they were not included (you have deleted them) but in the discussion it appears that you intended to include them but indeed there were no preterm infants that met your inclusion criteria. Please clarify. My suggestion is that you leave preterm infants in as an inclusion criteria, but state that none met the inclusion criteria. The discussion could then be about the implications of not having preterms in the study

Response: Sorry for the unclear messages. Yes, we intended to include the preterm infants but ended up with no preterm infants included in the study due to none was met the inclusion criteria as you understood. The Participants and Discussion sections were revised accordingly.

Editor: 5. Table 1. As suggested by the statistical reviewer please remove the statistics. In my view you don’t need all the columns – only the column with totals. However some of the data on the table are not actually baseline characteristics. For example formula use before 24, at 24-48 and 48-72 hours. Consider whether these need to go into a separate table. To avoid confusion please also change the data collection times to hours.

Response: Thank you for your suggestion. The statistics portion of Table 1 was removed as suggested. The data, which were not actually baseline characteristics, were separated to Table 2. The data collection unit was changed from times to hours as advised.

Editor: 6. Statistical tests. The wording still suggests that both tests (ANOVA and Kruskal Wallace) were used simultaneously. Is it due to the word ‘’and’’. Don’t forget to take the footnote out of Table 1 since you will be taking out the statistical data.

Response: Since the Kruskal Wallis test was used for continuous data with non-normal distribution, the Kruskal Wallis test was applied for monthly income and neonatal birthweight in Table 1. After removing the statistics in Table 1 as suggested, the Kruskal Wallis test was not used in remaining statistical tests. Thus, the Statistical analysis section was revised to remove the Kruskal Wallis test accordingly.

Editor: 7 & 8. Adverse drug effects. It would be helpful to give more information about how adverse drug reactions were sought. Specifically were the participants asked directly about each of the outcomes listed or were they asked a broad question such as do you have any other symptoms. It may be that this is what was listed is just the authors definitions of what would be considered an adverse event. This would clear up the confusion about Table 2. Adverse effects are still not clear from the table. While I understand that you collected adverse events at each 24-hour period the table does suggest there were 3 adverse events in the tea group and 3 in the domperidone group - one presenting in each 24-hour period. This needs to be made clearer.

Response: Each of the drug adverse events including headache, dry mouth, diarrhea, muscle cramps, itching, or allergic reactions was asked one by one. The previous data in Table 2 were intended to suggest that one adverse event (diarrhea) in tea group persisted for three days and one adverse event (dry mouth) in domperidone group persisted for three days. We agree that the previous data in Table was not clear enough. Hence, the new Table 3 (previously Table 2) was revised to deliver clear message of adverse event by report the adverse event only at the time of onset to reduce misunderstanding.

Editor: 9. Methods: I think more detail is needed on the feeding policy of babies after caesarean birth. I note from the methods that the first feeding appears to be at 12-18 hours after birth. Is this correct and if so is this the standard procedure? Most hospitals would start skin to skin and allow breastfeeding either in the operation theatre delivery is under spinal or epidural or as soon as the mother was awake when given general anaesthesia. So for many readers it is unclear whether the baby had been allowed to breastfeed prior to recruitment. Were babies allowed to feed prior to recruitment at 12-18 hours and if so what feeding was allowed? Were mothers allowed to express milk before 12-18 hours?

Response: Sorry for miscommunication with the previous information. Both group has similar breastfeeding support for early breastfeeding, frequent breast feeding, correct positioning, and avoiding the use of formula when possible. The standard of care at the Sunpasithiprasong Hospital for early breastfeeding is as early as possible. However, the actual time in some cases might be as late as 24 hours after birth which might not match with the first intervention timepoint in this study. Thus, we promote early breastfeeding as possible and ensure that at least the first breastfeeding was done after the first dose of intervention which occurred 12-18 hours after birth but earlier breastfeeding would be better and there was no restriction on early breastfeeding prior 12-18 hours after birth. The Methods section was revised to clarify the miscommunication.

Editor: 10. Results: Adding a line into the text to indicate that 'All participants completed their prescribed intervention and were analysed based on their randomization group' will help to reduce future queries on drop-outs. (If this is true)

Response: Thank you for your suggestion. This statement was true and was added in the Statistical analysis section accordingly.

Editor: 11. Results: Reviewer 2 has a concern about statistical test results: The authors mention that going by the p values, there is no significant difference between the domperidone and placebo group. However, just looking at the p values does not make logical sense to me. If Wang Nam Yen Herbal tea was better than placebo and Wang Nam Yen Herbal tea is equal to Domperidone, therefore logically Domperidone should be better than placebo. I think it is very misleading to portray that domperidone is similar to placebo as it currently stands. What is the reason for this? Readers could interpret this as selective reporting bias.

Response: Thank you for pointing out this important concern. There was no particular reason for reporting that the domperidone is similar to placebo as the authors also believed that this similarity was due to use of Bonferroni correction, which was conservative method of post-hoc correction, thus we discussed this specific issue on Discussion section. However, the interpretation of result was impartial and followed the proper interpretation of Bonferroni correction. On the other hand, we concerned that if we did not interpret the data as the results presented, that might be another type of selective reporting bias. To be more specific in this study, the effect of herbal vs placebo was slightly higher than effect of domperidone vs placebo, thus it was not uncommon that while herbal vs domperidone was similar, the result of herbal vs placebo and result of domperidone vs placebo might not be the same when the statistical tests applied. This should not be interpreted that the domperidone was similar to placebo since the primary outcome of this study was to study the efficacy of herbal tea vs domperidone and herbal tea vs placebo. The manuscript text about the breast milk per expression at 72 hours postpartum for the domperidone group and the placebo group was deleted to reduce the confusion and to reflect the primary outcome while the result was still provided in the Table 3 to avoid the selective reporting bias.

Editor: 12. There are still many grammatical issues right through the manuscript. Reviewer 2 has pointed out a few. Please do get it read by someone with a good grasp of English.

Response: Thank you for your suggestion. The grammatical issues were revised throughout the manuscript accordingly.

We hope that our responses are satisfactory. Should there be anything that might improve our work, please kindly inform us. Thank you very much for your kind consideration.

Best Regards,

Assoc. Prof. Dr. Krit Pongpirul, MD, MPH, PhD.

On behalf of the authors
