# Supplementary material for: Efficacy of Wang Nam Yen herbal tea on human milk production: A randomized controlled trial
Source: PLoS One. 2022 Jan 31;17(1):e0247637. doi: 10.1371/journal.pone.0247637 (PMC8803155; doi:10.1371/journal.pone.0247637)
Supplement: Attachment — Submitted filename: responses.docx [file pone.0247637.s008.docx]

Dear Editor,

Please consider our response to your comment:

Editor: There is just one small issue and that is that there is no description on how adverse effects were collected. We do not know how the participant was asked. For example was the participant asked whether they experienced any adverse effects and if so what were they or was the participant asked specifically about whether they had experienced any of the listed adverse effects. If so where did this list come from? Were these know adverse effects of the intervention? If they were asked specifically about these was there also a question on whether they experienced any unexpected adverse effects. If they were asked about unexpected adverse effects and reported these what were they?

Response: Many thanks for the thorough assessment and the important comment. Each participant was asked for the presence of each of the potential adverse drug reactions to the study drugs, along with the post-partum clinical parameters, by the study nurse as described in the manuscript. Based on the findings from previous unpublished quasi-experimental study comparing the herbal tea (study group) with a pandan leave tea (control group) (Ref 18), participant in the study group reported no adverse drug reactions whereas one participant in the control group who had consumed the pandan leave tea for two days reported a 5-minute headache episode at 2-3 minutes after consuming the herbal tea on day 3 and day 4. As ‘headache’ could be a general subjective feeling after consuming tea of any kind, we merged the headache in the initial list of potential adverse drug reactions, along with that of domperidone as presented in the Methods section. The participants were also asked about any other symptoms that they experienced after consuming the study drugs that were not in the initial list. In fact, some participants reported a mild degree of abdominal discomfort and sleep difficulty but both symptoms were assessed by the investigators as not related to the study drugs.

We hope that our response is satisfactory. Should there be anything that might improve our work, please kindly inform us. Thank you very much for your kind consideration.

Best Regards,

Assoc. Prof. Dr. Krit Pongpirul, MD, MPH, PhD.

On behalf of the authors
